# Phylogenetic analyses allow species-level recognition of *Leptographium wageneri* varieties that cause black stain root disease of conifers in western North America

**DOI:** 10.3389/fpls.2023.1286157

**Published:** 2023-12-22

**Authors:** Daram Choi, Thomas C. Harrington, David C. Shaw, Jane E. Stewart, Ned B. Klopfenstein, Duncan R. Kroese, Mee-Sook Kim

**Affiliations:** ^1^Department of Forest Engineering, Resources and Management, Oregon State University, Corvallis, OR, United States; ^2^Pacific Northwest Research Station, USDA Forest Service, Corvallis, OR, United States; ^3^Department of Plant Pathology, Entomology and Microbiology, Iowa State University, Ames, IA, United States; ^4^Department of Agricultural Biology, Colorado State University, Fort Collins, CO, United States; ^5^Rocky Mountain Research Station, USDA Forest Service, Moscow, ID, United States

**Keywords:** *Leptographium*, phylogeny, genetic characterization, forest pathogen, taxonomy

## Abstract

*Leptographium wageneri* is a native fungal pathogen in western North America that causes black stain root disease (BSRD) of conifers. Three host-specialized varieties of this pathogen were previously described: *L. wageneri* var. *wageneri* on pinyon pines (*Pinus monophylla* and *P*. *edulis*); *L. wageneri* var. *ponderosum*, primarily on hard pines (e.g., *P*. *ponderosa*, *P*. *jeffreyi*); and *L. wageneri* var. *pseudotsugae* on Douglas-fir (*Pseudotsuga menziesii*). Morphological, physiological, and ecological differences among the three pathogen varieties have been previously determined; however, DNA-based characterization and analyses are needed to determine the genetic relationships among these varieties. The objective of this study was to use DNA sequences of 10 gene regions to assess phylogenetic relationships among *L. wageneri* isolates collected from different hosts. The multigene phylogenetic analyses, based on maximum likelihood and Bayesian inference, strongly supported species-level separation of the three *L. wageneri* varieties. These results, in conjunction with previously established phenotypic differences, support the elevation of *L. wageneri* var. *ponderosum* and *L. wageneri* var. *pseudotsugae* to the species level as *L*. *ponderosum* comb. nov. and *L*. *pseudotsugae* comb. nov., respectively, while maintaining *L. wageneri* var. *wageneri* as *Leptographium wageneri*. Characterization of the three *Leptographium* species, each with distinct host ranges, provides a baseline to further understand the ecological interactions and evolutionary relationships of these forest pathogens, which informs management of black stain root disease.

## Introduction

Black stain root disease (BSRD) is a vascular wilt disease of conifers that is damaging to forests in western North America, especially in the western United States (Washington, Oregon, and California), and southwestern Canada (British Columbia) (Lockman and Kearns, 2016). BSRD was first found producing a dark sapwood stain in hard pines [Jeffrey pine (*Pinus jeffreyi*) and ponderosa pine (*P. ponderosa*)] and single-leaf pinyon (*P. monophylla*) in 1938 and 1941, respectively, in California (Wagener and Mielke, 1961). Although BSRD was not well recognized in the Pacific Northwest of United States before 1969, it has emerged as one of the five most-damaging root diseases in western forests of the United States (Hadfield et al., 1986; Cobb, 1988). The disease is caused by a native, insect-vectored ascomycete fungal pathogen, originally named *Verticladiella wageneri* W.B Kendr. (Kendrick, 1962) and later transferred to *Leptographium wageneri* (W.B Kendr.) M.J. Wingf. (Wingfield, 1985).

**Figure 1:**
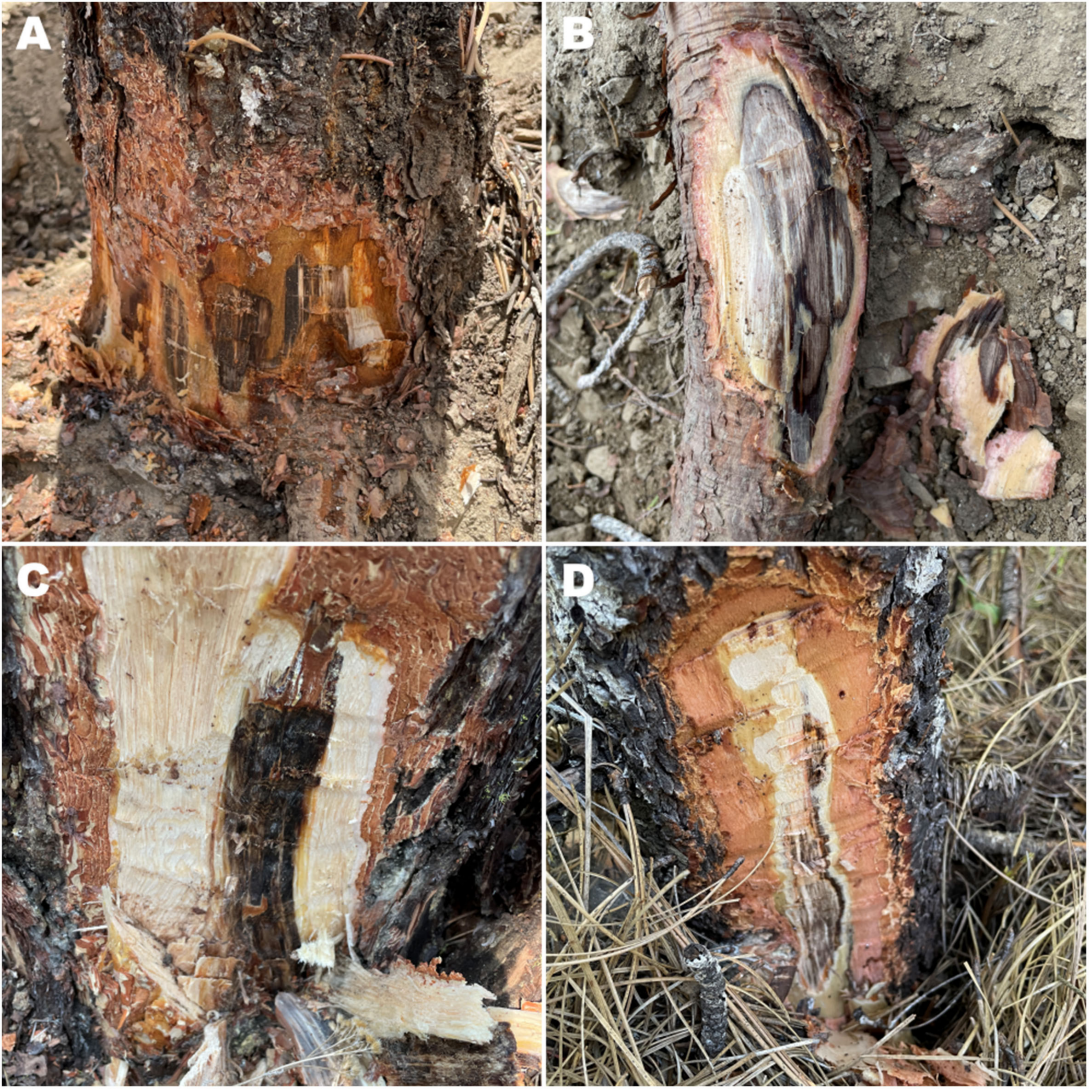
Black streaking of sapwood, which is associated with black stain root disease (caused by *Leptographium wageneri* varieties s.l.), appears in **(A)** root collars above ground and **(B)** underground roots from a single-leaf pinyon (*Pinus monophylla*). Staining appears above ground level in **(C)** ponderosa pine (*P. ponderosa*) and **(D)** Douglas-fir (*Pseudotsuga menziesii*).

*Leptographium wageneri* can be introduced to hosts in two ways: 1) via root contacts or grafts between healthy and infected trees, or 2) via insect vectors. Wounds may be required for non-root graft infection because the pathogen’s hyphae cannot penetrate bark or break down cellulose (Hessburg, 1984), though small roots may be infected directly (Cobb, 1988). Once the BSRD fungal pathogen enters the host, it colonizes tracheid cells in sapwood xylem, and dark-brown to purple-black staining starts to appear when the xylem is colonized by hyphae (**Figure 1**) (Wagener and Mielke, 1961; Hessburg et al., 1995). Other than the characteristic staining, infected hosts often show general wilt disease symptoms, such as tufted needles, needle loss, chlorosis, and/or reduced growth (**Supplementary Figure 1**), typically followed by tree mortality. Basal resin flow may also occur, and a stress cone crop may be produced in Douglas-fir (*Pseudotsuga menziesii*). Because the crown symptoms are similar to other root diseases, the best way to confirm BSRD in the field is by verifying the presence of the dark stains in sapwood of roots and/or at the base of the stem (**Figure 1**) (Hadfield et al., 1986).

The sexual stage of the BSRD pathogen was described as *Ceratocystis wageneri* Goheen & F.W. Cobb [syn. *Ophiostoma wageneri* (Goheen & F.W. Cobb) T.C. Harr. & F.W. Cobb; syn, *Grosmannia wageneri* (Goheen & F.W. Cobb) Zipfel, Z.W. de Beer & M.J. Wingf.] (Goheen and Cobb, 1978; Harrington and Cobb, 1987; Zipfel et al., 2005). Perithecia and ascospores were found in an insect gallery in a root of a ponderosa pine in a diseased stand in California (Goheen and Cobb, 1978), but no culture of the teleomorph is available, and subsequent searches in this same location failed to find perithecia of *L. wageneri* (Harrington, 1988). No further reports of a sexual stage have been made. Further, the sexual stage of other heterothallic *Leptographium* species are readily produced in culture if opposite mating types are paired (Duong et al., 2016), but pairing of isolates from ponderosa pine and other hosts failed to produce perithecia (Harrington and Cobb, 1986).

In culture, the BSRD pathogens produce stalked conidiophores with sticky drops of conidia, which appear suitable for insect dispersal, and such conidia are presumably formed in galleries produced by the insect vectors. When young, adult insects emerge from infected roots, their exoskeleton may be contaminated by conidia. These conidia can be subsequently introduced to roots of healthy trees when these adult insect vectors bore into roots for egg laying or maturation feeding (Hessburg et al., 1995; Ferguson, 2009). Root-feeding insects, such as bark beetles (*Hylastes nigrinus* and *H. macer*) and weevils (*Pissodes fasciatus* and *Stereminius carinatus*) (Coleoptera: Curculionidae), are the primary vectors involved in spread of the BSRD pathogen (Hessburg et al., 1995; Ferguson, 2009). Among these potential vectors, the conifer seedling weevil, *S. carinatus*, is considered an important agent for within stand spread because it is flightless (Hadfield et al., 1986; Ferguson, 2009).

*Leptographium wageneri* was known to have three varieties: *L. wageneri* var. *ponderosum*, primarily on ponderosa, Jeffrey, and lodgepole (*P*. *contorta*) pines; *L. wageneri* var. *pseudotsugae*, primarily on Douglas-fir; and the originally described species (*L. wageneri* var. *wageneri*), a pathogen on single-leaf pinyon and pinyon pines (*P. edulis*) (Harrington and Cobb, 1986). These three varieties can be distinguished by their distinct range of host preferences (Harrington and Cobb, 1984), minor morphological and physiological differences (Harrington and Cobb, 1987), and isozyme variation (Zambino and Harrington, 1989; Zambino and Harrington, 1992).

Isozyme variation and random amplified polymorphic DNA (RAPD) have previously distinguished the three varieties of *L. wageneri* (Otrosina and Cobb, 1987; Zambino and Harrington, 1989; Zambino and Harrington, 1992; Witthuhn et al., 1997), and population genomics have been used to distinguish differences between two varieties, *L. wageneri* var. *wageneri* and *L. wageneri* var. *pseudotsugae* (Bennett et al., 2021). Previous studies have suggested that very limited genetic variation is found within *L. wageneri*. Few genetic loci have been sequenced for isolates of *L. wageneri*, and limited numbers of sequences are publicly available (National Center for Biotechnology Information; NCBI, GenBank, https://www.ncbi.nlm.nih.gov/genbank/), including some sequences for rDNA [e.g., internal transcribed spacer (ITS) regions, 28S (large subunit, LSU), and 18S (small subunit, SSU)], actin (*ACT*), β-tubulin (*TUB*), calmodulin (*CAL*), elongation factor-1alpha (*TEF-1α*) gene, mating type genes (*MAT1-2-1* and *MAT1-1-3*), and RNA polymerase II (*RPB2*) (Jacobs et al., 2001; Hausner et al., 2005; Zipfel et al., 2005; Duong et al., 2016; Marincowitz et al., 2017; Bennett et al., 2021; de Beer et al., 2022). Some of these deposited sequences are from isolates of unknown geographic and host origin, which could provide information to help identify the *L. wageneri* variety. In every locus except *TUB*, only a few isolates for each *L. wageneri* variety are available. Other phylogenetic studies of *Leptographium* and related species (e.g., *Grosmannia*) have used DNA sequences from a limited number of *L. wageneri* isolates representing several loci, such as the ITS region 2 (ITS2) and LSU rDNA, *ACT*, *TUB*, *CAL*, and *TEF-1α* (Duong et al., 2012; Linnakoski et al., 2012; Yin et al., 2015). Because the ITS region is difficult to amplify and sequence for *L. wageneri* s.l. and related fungi, the ITS region has not been widely used as a barcoding region for distinguishing among some Ophiostomatales (Witthuhn et al., 1997; Harrington et al., 2010).

We hypothesized that the three varieties of *L. wageneri* are phylogenetically distinct and genetic differences are sufficient to warrant their recognition at the species level. Thorough phylogenetic analyses of *L. wageneri* requires ample isolates and accurate sequences from a sufficient number of diverse loci. The overall objective of this study was to use DNA sequences derived from multiple loci to genetically characterize the three *L. wageneri* varieties collected from western North America and determine their phylogenetic relationships.

## Materials and methods

### Study sites and sample collection

During 2019-2021, a total of 24 BSRD samples were collected from various National Forests where BSRD was previously reported in Idaho, Oregon, and California (**Supplementary Table 1**). Samples were collected from three single-leaf pinyon pines, six ponderosa pines, two Jeffrey pines, and nine Douglas-fir trees. The number of samples collected from each host species varied depending on site accessibility, disease incidence, and previous records. Trees with BSRD crown symptoms were examined for black or dark-brown streaks in the sapwood near the roots and/or basal stems. For each tree, three to five wood samples (ca. 2 x 7 x 1 cm) were collected directly from the stained tissue and stored in a sealed plastic bag assigned to each tree with paper towels dampened in sterile water. Samples were kept cool by storage on ice or refrigeration before fungal isolation was performed. Precise locations were collected using a GPSMAP 64s GPS receiver (Garmin, Olathe, KS, USA). Diameter at breast height (DBH), health status (dead/live/declining and crown status), and other noticeable field conditions (e.g., elevation, vegetation, stand conditions) were also recorded. Two-thirds of the collected samples were sent to USDA Forest Service – Pacific Northwest Research Station, Forest Heath Laboratory (Corvallis, OR) for isolation within 48 hours of collection, and one-third of samples were isolated on-site.

### Sample isolation

For surface disinfestation, the wood samples were cut into smaller pieces containing mostly stained areas (**Supplementary Figure 2**). The wood pieces were submerged and soaked in 95% ethanol for 30 seconds, and transferred to 10% commercial bleach (0.05% sodium hypochlorite) for a 3-minute soak. The samples were then rinsed four times in a separate beaker with sterile, deionized H_2_O. Damp surfaces were dried with sterile tissues, and the samples were cut into pieces (ca. 10 x 2.5 x 2.5 mm) for culturing. Approximately ten pieces were embedded in a Petri dish containing a selective medium with malt extract agar (MEA) amended with cycloheximide and streptomycin sulfate (CSMA) (one-fourth strength MEA media: 0.75% malt, 0.75% dextrose, 0.25% peptone, and 1.5% agar with 200 ppm cycloheximide and 200 ppm streptomycin sulfate) (Harrington, 1992). Three to five Petri dishes were established for samples from each tree. During incubation, samples were stored at 15°C, checked daily, and discarded if contamination occurred. After 2-3 weeks, when *L. wageneri* conidiophores were observed on the wood piece, drops of conidia were transferred from the conidiophores onto MEA medium (3% malt, 3% dextrose, 1% peptone, 1.5% agar). After 1-2 weeks of incubation, when fungal hyphae started to grow from the transferred conidia, hyphal tips were transferred onto fresh MEA medium with the use of a flame-sterilized dissecting pin. In addition, 15 isolates were studied from collections made in the 1980s (Harrington and Cobb, 1986; Harrington and Cobb, 1987). These previously collected isolates were used in descriptions of the varieties, including ex-types (CAP-19 and CAD-18), and they are now archived at the USDA Forest Service – Pacific Northwest Research Station, Forest Heath Laboratory (Corvallis, OR) (**Supplementary Table 1**).

### DNA extraction and PCR

For DNA extractions, a total of 39 *L. wageneri* hyphal-tipped isolates were grown on 0.2-μm-pore, nylon-membrane filters (Millipore Corporation, Burlington, MA, USA) overlaid on MEA medium and grown at 15°C for 2-3 weeks. Mycelia, ca. 50-100 mg, were scraped directly from the filters and homogenized in extraction buffer (BashingBead™ Buffer, Zymo Research, Irvine, CA, USA) using FastPrep-24™ 5G (MP Biomedicals, Santa Ana, CA, USA). DNA extraction was conducted according to the ZR Fungal/Bacterial DNA MiniPrep Kit protocols supplied by Zymo Research (Irvine, CA, USA). DNA concentration was measured using a NanoDrop™ 2000 Spectrophotometer (Thermo Fisher Scientific, Waltham, MA, USA). Species identification of *L. wageneri* s.l. isolates was confirmed by comparisons with *TUB* sequences in GenBank using BLAST (Basic Local Alignment Search Tool) (http://blast.ncbi.nlm.nih.gov/Blast.cgi).

Polymerase Chain Reaction (PCR) was used to amplify the following ten loci for sequencing: 28S LSU, ACT* (primers Lepact-F & Lepact-R) and ACT** (primers ACT512F & ACT783R), *TUB*, *CAL*, *TEF-1α*, a mating-type gene (*MAT1-1-3*), *RPB2*, glyceraldehyde-3-phosphate dehydrogenase (*GPD*), and chitin synthase (*CHS*). Two regions of *ACT* were amplified using two different primer sets, and they are differentiated as ACT* and ACT** (**Supplementary Table 2**).

The PCR mixture contained 4 µL template DNA (ca. 25ng/µL), 5 µL 10X PCR reaction buffer, 1 µL dNTP, 5 µL (5 nM) of each primer, and 0.5 µL (2.5 units) Green Taq DNA polymerase (GenScript Biotech, Piscataway, NJ, USA) in a total volume of 50 µL. The PCR reaction was conducted using a C1000 Touch ™ Thermal Cycler (Bio-Rad, Hercules, CA, USA), and the thermocycling setting for each primer pair followed the published references with minor modifications (**Supplementary Table 3**). The peak sizes of the PCR amplicons were determined using an automated electrophoresis analyzer, QIAxcel^®^ Advanced (QIAGEN, Hilden, Germany). Amplified PCR products were then purified with ExoSAP-IT™ Express (Thermo Fisher Scientific, Waltham, MA, USA), following the manufacturer-suggested protocol. Purified PCR products were sent to Psomagen, Inc. (Rockville, MD, USA) for Sanger sequencing.

### Phylogenetic analyses

The resulting sequences of each *L. wageneri* s.l. isolate consisted of forward and reverse ABI sequencer data files (.ab1). Geneious Prime version 2022.2.2 (https://www.geneious.com/) was used to pair and edit the sequences following IUPAC (International Union of Pure and Applied Chemistry) codes. The 39 paired sequences for each of the 10 loci were generated as consensus sequences, and they were aligned using Clustal Omega 1.2.2. (Sievers et al., 2011) with default parameters and trimmed to the same length for comparison.

The sequences of the loci were analyzed in four parts: Analysis 1) individual loci; Analysis 2) concatenated sequences of two (*CAL* + ACT*) and three (*CAL* + ACT* + *TEF-1α*) loci; Analysis 3) concatenated sequences of 10 loci; and Analysis 4) concatenated sequences of two loci (*CAL* + ACT*) analyzed with multiple outgroups (**Supplementary Table 4**). *Leptographium douglasii* was used as an outgroup for single outgroup analyses (Analysis 1, 2, and 3). The sequences of ACT**, *GPD*, and *CHS* of *L. douglasii* were not available in GenBank, therefore, these sequences were generated using the same primer sets and protocols as for *L. wageneri* s.l. For Analysis 2, the two- and three-loci combinations were selected because each of the loci have relatively large numbers of parsimony-informative sites. Parsimony-informative sites were identified using IQ-TREE (Nguyen et al., 2014). For Analysis 4, six outgroup taxa (*L. douglasii*, *L. rhodanense, L. gracile, L. castellanum, G. alacris*, and *G. serpens*) were selected based on the phylogenetic tree from de Beer et al. (2022) and availability of sequences in GenBank.

A partition homogeneity test was performed using PAUP* version 4.0a169 (Swofford and Sullivan, 2003) to determine whether the sequence data for the 10 loci were congruent for concatenation. A p-value over 0.05 indicates that partitions can be combined, and the sequence data of the 10 loci resulted in a p-value = 0.08. The best-fit substitution model for each locus was selected by Bayesian Information Criterion (BIC) calculated in ModelFinder in IQ-TREE (Nguyen et al., 2014; Kalyaanamoorthy et al., 2017) (**Supplementary Table 5**). Phylogenetic analyses were implemented using maximum likelihood and Bayesian inference. Maximum likelihood trees were constructed using IQ-TREE (Nguyen et al., 2014; Hoang et al., 2018) with 1,000 bootstrap pseudo-replications. Maximum likelihood trees were imported and visualized using Geneious Prime, with bootstrap support values (BS) over 50%. MrBayes (Huelsenbeck and Ronquist, 2001) plugin in Geneious Prime was used for the Bayesian inference. Because Tamura–Nei (TN) models could not be implemented using MrBayes in Geneious Prime, the next best models were selected. Bayesian trees were generated using 1,100,000 generations with the four heated-chains setting in Markov Chain Monte Carlo (MCMC). The first 100,000 chains were discarded as burn-in, posterior probability (PP) for each dataset was calculated, and a consensus tree was produced with PP over 0.80 illustrated on the branches.

## Results

Sequences from 39 *L. wageneri* s.l. isolates (eight, 14, and 17 for *L. wageneri* var. *wageneri*, *L. wageneri* var. *ponderosum*, and *L. wageneri* var. *pseudotsugae*, respectively) were obtained for the 10 loci (**Table 1**).

**Table 1:** GenBank accession numbers and host information for 8 *Leptographium wageneri* (formerly *L*. *wageneri* var. *wageneri*), 14 *L. ponderosum* (formerly *L*. *wageneri* var. *ponderosum*), and 17 *L. pseudotsugae* (formerly *L*. *wageneri* var. *pseudotsugae*) isolates for 10 loci [28S large subunit rDNA (LSU), actin (ACT*; Lepact-F & Lepact-R), actin (ACT**; ACT512F & ACT783R), β-tubulin (*TUB*), calmodulin (*CAL*), elongation factor-1alpha (*TEF-1α*) gene, a mating-type gene (*MAT 1-1-3*), RNA polymerase II (*RPB2*), glyceraldehyde-3-phosphate dehydrogenase (*GPD*), and chitin synthase (*CHS*)].

Sample ID	Host	State or Provence of Origin	GenBank Accession Number									
			28S LSU	ACT*	ACT**	*TUB*	*CAL*	*TEF-1α*	*MAT1-1-3*	*RPB2*	*GPD*	*CHS*
*Leptographium wageneri (formerly L. wageneri var. wageneri)*												
CA_PIMO_3	*Pinus monophylla*	California	OR479138	OR499888	OR499900	OR400356	OR479164	OR479174	OR479150	OR494471	OR506896	OR479186
CA_PIMO_4**^†^**	*Pinus monophylla*	California	–	–	–	–	–	–	–	–	–	–
CA_PIMO_7**^†^**	*Pinus monophylla*	California	–	–	–	–	–	–	–	–	–	–
CAS-1	*Pinus monophylla*	California	OR479133	OR499893	OR499905	OR400360	OR479162	OR479169	OR479145	OR494476	OR506901	OR479181
CAS-2**^†^**	*Pinus monophylla*	California	OR479132	OR499894	OR499906	OR400361	OR479161	OR479168	OR479144	OR494477	OR506902	OR479180
CAS-15**^†^**	*Pinus monophylla*	California	–	–	–	–	–	–	–	–	–	–
NES-1**^†^**	*Pinus monophylla*	Nevada	–	–	–	–	–	–	–	–	–	–
NME-1**^†^**	*Pinus edulis*	New Mexico	–	–	–	–	–	–	–	–	–	–
*Leptographium ponderosum (formerly L. wageneri var. ponderosum)*												
OR_PIPO_2**^†^**	*Pinus ponderosa*	Oregon	–	–	–	–	–	–	–	–	–	–
CA_PIPO_9**^†^**	*Pinus ponderosa*	California	–	–	–	–	–	–	–	–	–	–
CA_PIPO_11**^†^**	*Pinus ponderosa*	California	–	–	–	–	–	–	–	–	–	–
CA_PIPO_15**^†^**	*Pinus ponderosa*	California	–	–	–	–	–	–	–	–	–	–
CA_PIPO_28**^†^**	*Pinus ponderosa*	California	–	–	–	–	–	–	–	–	–	–
CA_PIPO_34**^†^**	*Pinus ponderosa*	California	–	–	–	–	–	–	–	–	–	–
CA_PIJE_7**^†^**	*Pinus jeffreyi*	California	–	–	–	–	–	–	–	–	–	–
CA_PIJE_11**^†^**	*Pinus jeffreyi*	California	–	–	–	–	–	–	–	–	–	–
BCL-1**^†^**	*Pinus contorta*	British Columbia	–	–	–	–	–	–	–	–	–	–
ORH-1**^†^**	*Tsuga heterophylla*	Oregon	–	–	–	–	–	–	–	–	–	–
ORM-S**^†^**	*Tsuga mertensiana*	Oregon	–	–	–	–	–	–	–	–	–	–
IDP-1**^†^**	*Pinus ponderosa*	Idaho	–	–	–	–	–	–	–	–	–	–
CAP-19**^†^**	*Pinus ponderosa*	California	OR479134	OR499892	OR499904	OR400353	OR479163	OR479170	OR479146	OR494475	OR506900	OR479182
CA_PIPO_5**^†^**	*Pinus ponderosa*	California	–	–	–	–	–	–	–	–	–	–
*Leptographium pseudotsugae (formerly L. wageneri var. pseudotsugae)*												
CA_PSME_6**^†^**	*Pseudotsuga menziesii*	California	–	–	–	–	–	–	–	–	–	–
CA_PSME_8**^†^**	*Pseudotsuga menziesii*	California	–	–	–	–	–	–	–	–	–	–
CA_PSME_9**^†^**	*Pseudotsuga menziesii*	California	–	–	–	–	–	–	–	–	–	–
CA_PSME_10	*Pseudotsuga menziesii*	California	OR479137	OR499889	OR499901	OR400357	OR479157	OR479173	OR479149	OR494472	OR506897	OR479185
CA_PSME_11**^†^**	*Pseudotsuga menziesii*	California	–	–	–	–	–	–	–	–	–	–
CA_PSME_12**^†^**	*Pseudotsuga menziesii*	California	–	–	–	–	–	–	–	–	–	–
CA_PSME_13**^†^**	*Pseudotsuga menziesii*	California	–	–	–	–	–	–	–	–	–	–
CA_PSME_14	*Pseudotsuga menziesii*	California	OR479136	OR499890	OR499902	OR400358	OR479156	OR479172	OR479148	OR494473	OR506898	OR479184
CA_PSME_15**^†^**	*Pseudotsuga menziesii*	California	–	–	–	–	–	–	–	–	–	–
BCH-1	*Tsuga heterophylla*	British Columbia	OR479139	OR499887	OR499899	OR400355	OR479158	OR479175	OR479151	OR494470	OR506895	OR479187
BCD-1	*Pseudotsuga menziesii*	British Columbia	OR479140	OR499886	OR499898	OR400354	OR479159	OR479176	OR479152	OR494469	OR506894	OR479188
CAD-18**^†^**	*Pseudotsuga menziesii*	California	OR479135	OR499891	OR499903	OR400359	OR479155	OR479171	OR479147	OR494474	OR506899	OR479183
COD-2	*Pseudotsuga menziesii*	Colorado	OR479131	OR499895	OR499907	OR400362	OR479160	OR479167	OR479143	OR494478	OR506903	OR479179
IDD-1	*Pseudotsuga menziesii*	Idaho	OR479129	OR499897	OR499909	OR400364	OR479153	OR479165	OR479141	OR494480	OR506905	OR479177
ID_PSME_1	*Pseudotsuga menziesii*	Idaho	OR479130	OR499896	OR499908	OR400363	OR479154	OR479166	OR479142	OR494479	OR506904	OR479178
CA_PSME_1**^†^**	*Pseudotsuga menziesii*	California	–	–	–	–	–	–	–	–	–	–
CA_PSME_3**^†^**	*Pseudotsuga menziesii*	California	–	–	–	–	–	–	–	–	–	–

Dagger (^†^) indicates that isolates are identical within each species. Underlined isolates (CAP-19 and CAD-18) are ex-type isolate of each species.

### Individual loci

No parsimony-informative sites were found among the three *L. wageneri* varieties at the LSU, *TUB*, and *RPB2* loci. The sequences of LSU and *RPB2* were identical among the 39 isolates, and only a single isolate of *L. wageneri* var. *ponderosum* showed a single base substitution for *TUB*.

One variety, *L. wageneri* var. *ponderosum*, was separated from the other two varieties based on single-base substitutions in the sequences for ACT** (**Supplementary Figure 3**), *MAT1-1-3* (**Supplementary Figure 4**), *GPD* (**Supplementary Figure 5**), and *CHS* (**Supplementary Figure 6**) (with 95.49%, 99.33%, 99.63%, and 98.53% constant sites, respectively). In the ACT** tree, *L. wageneri* var. *pseudotsugae* and var. *wageneri* were separated from var. *ponderosum* (0.99 PP and 67 BS) with a few exceptions. In the *MAT1-1-3* tree, separation of *L. wageneri* var. *wageneri* isolates was well-supported (0.98 PP and 63 BS); however, isolate COD-2 (*L. wageneri* var. *pseudotsugae*) was included in the var. *wageneri* clade. The *GPD* tree showed a well-supported (0.99 PP and 61 BS) separation of *L. wageneri* var. *ponderosum*. In the *CHS* tree, *L. wageneri* var. *pseudotsugae* isolates, except COD-2, had identical sequences and were separated from the other varieties (0.99 PP and 65 BS); the *CHS* sequence of isolate COD-2 (*L. wageneri* var. *pseudotsugae*) grouped with the other two varieties at some loci.

**Figure 2:**
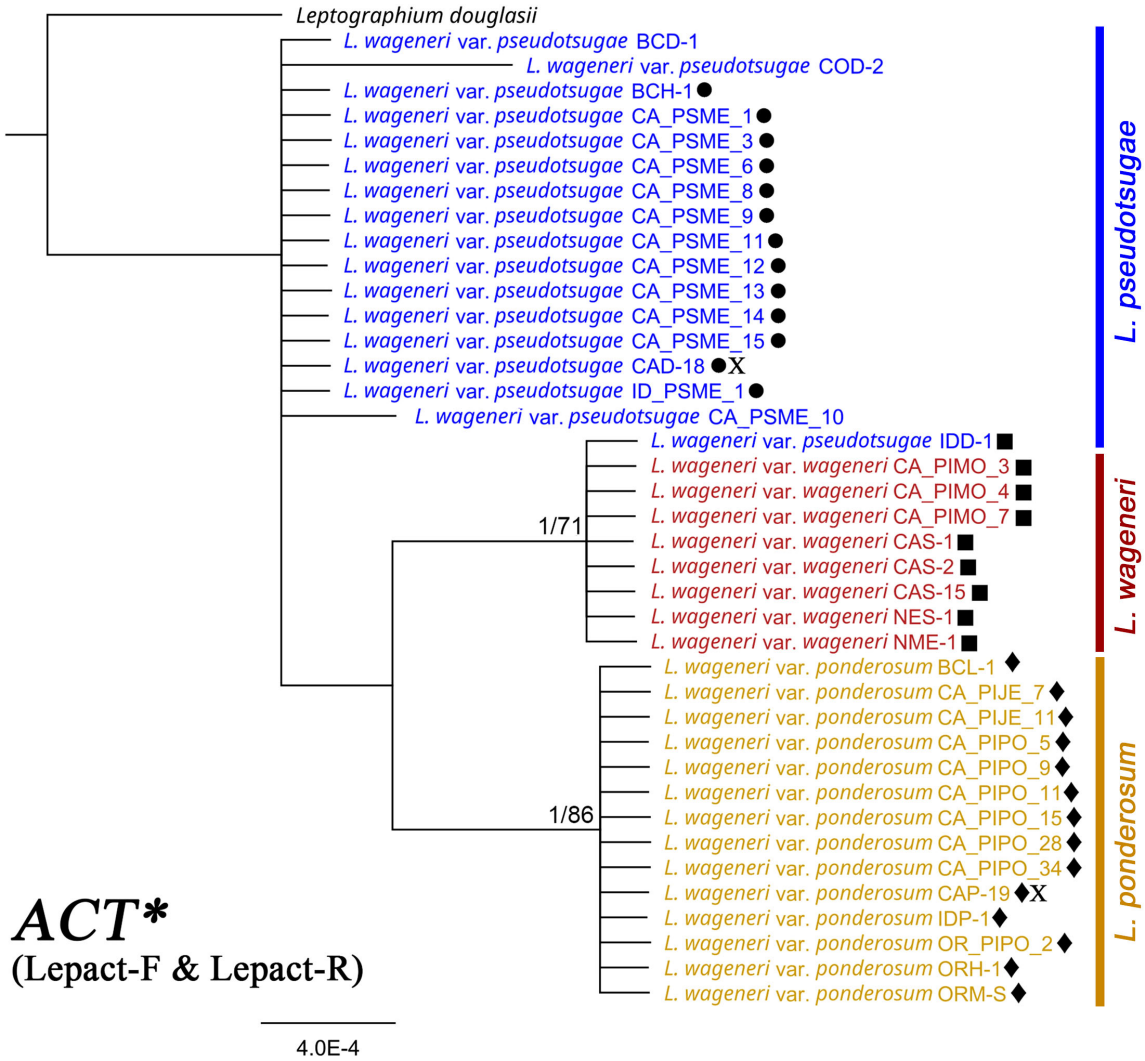
Phylogeny of actin (ACT*) with *Leptographium douglasii* (GenBank accession #KY424502) used as the outgroup. *Leptographium wageneri* (formerly *L*. *wageneri* var. *wageneri*), *L*. *ponderosum* (formerly *L. wageneri* var. *ponderosum*), and *L. pseudotsugae* (formerly *L. wageneri* var. *pseudotsugae*) are color-coded in red, yellow, and blue, respectively. The round, square, and diamond shapes inserted to the right of the isolate name indicate identical sequences. Numbers at each node indicate supports posterior probabilities (PP) greater than 0.80 and bootstrap support (BS) values greater than 50% (PP/BS). ACT* indicates actin dataset using Lepact-F & Lepact-R primers. Letter X indicates ex-type cultures of each variety.

Sequences of *CAL* (**Supplementary Figure 7**), ACT* (**Figure 2**), and *TEF-1α* (**Supplementary Figure 8**) contained nine, four, and two parsimony-informative sites, with 98.41%, 99.15%, and 99.12% constant sites, respectively. In the *CAL*-based tree, the clade of *L. wageneri* var. *ponderosum* and var. *wageneri* was well-supported (1 PP and 97 BS). In the ACT*-based tree, the clades of *L. wageneri* var. *ponderosum* and var. *wageneri* were well-supported (1 PP and 86 BS; 1 PP and 71 BS, respectively); however, isolate IDD-1 (*L. wageneri* var. *pseudotsugae*) was an exception and grouped with the var. *wageneri* clade. Out of the 10 individual loci, single locus-based separation of the three varieties was observed only for ACT*. The *TEF-1α*-based tree separated *L. wageneri* var. *wageneri* (0.99 PP and 72 BS) from the other varieties.

### Concatenation of two and three loci

Analyses using the combined data of the most polymorphic loci clearly separated the *L. wageneri* varieties. The three varieties were well separated in the tree generated with the two loci, *CAL* and ACT* (**Supplementary Figure 9**). The clade containing the *L. wageneri* var. *ponderosum* isolates was well-supported (0.97 PP and 88 BS), and a *L. wageneri* var. *pseudotsugae* clade was also supported (0.93 PP and 56 BS). The three varieties were also clearly separated based on concatenated sequences of *CAL*, ACT*, and *TEF-1α* (**Supplementary Figure 10**). In the phylogenetic tree based on the concatenated sequences of these three loci, each variety was well-supported: *L. wageneri* var. *wageneri* at 1 PP and 80 BS, var. *ponderosum* at 0.96 PP and 83 BS, and var. *pseudotsugae* at 0.96 PP and 63 BS.

### Concatenated of 10 loci

**Figure 3:**
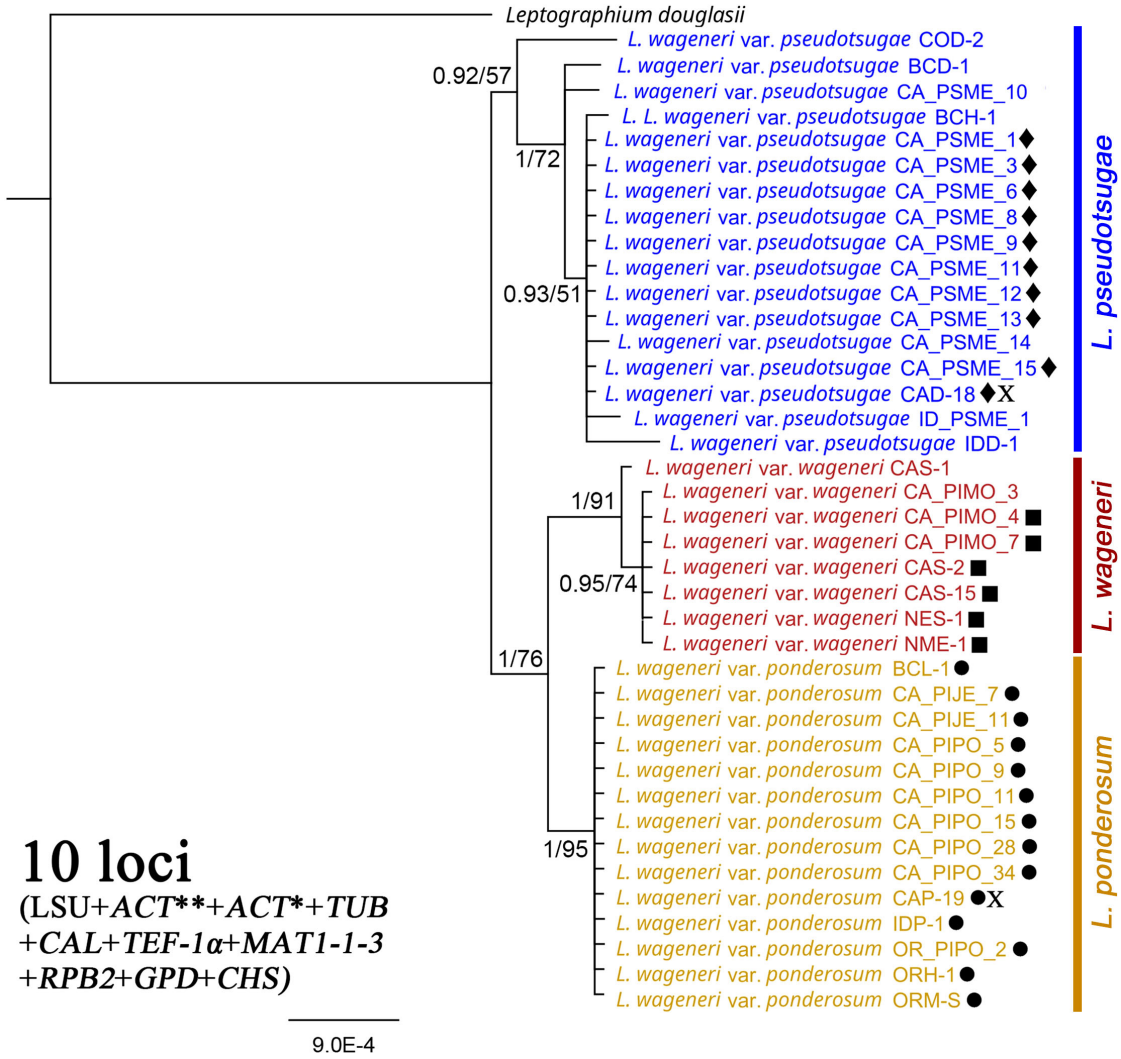
Phylogeny of the combined 28S large subunit (LSU) rDNA, actins (ACT** and ACT*), β-tubulin (TUB), calmodulin (CAL), translation elongation factor 1-alpha (*TEF-1α*), mating-type gene (*MAT1-1-3*), RNA polymerase II subunit (*RPB2*), glyceraldehyde-3-phosphate dehydrogenase (*GPD*), and chitin synthase (*CHS*) loci with *Leptographium douglasii* used as an outgroup. *Leptographium wageneri* (formerly *L*. *wageneri* var. *wageneri*), *L*. *ponderosum* (formerly *L. wageneri* var. *ponderosum*), and *L. pseudotsugae* (formerly *L. wageneri* var. *pseudotsugae*) are color-coded in red, yellow, and blue, respectively. The round, square, and diamond shapes inserted to the right of the isolate name indicate identical sequences. Numbers at each node indicate posterior probabilities (PP) greater than 0.80 and bootstrap support (BS) values greater than 50% (PP/BS). ACT* and ACT** indicate actin dataset using Lepact-F & Lepact-R and ACT512F & ACT783R primers, respectively. Letter X indicates ex-type cultures of each variety.

In the phylogenetic tree based on concatenated sequences of the 10 loci (LSU, *TUB*, *RPB2*, *MAT1-1-3*, *CAL*, ACT*, *TEF-1α*, ACT**, *GPD*, and *CHS*), each of the three varieties was well-supported as a separate clade: *L. wageneri* var. *wageneri* (1 PP and 91 BS), *L. wageneri* var. *ponderosum* (1 PP and 95 BS), and *L. wageneri* var. *pseudotsugae* (0.92 PP and 57 BS) (**Figure 3**).

### Concatenation of two loci with multiple outgroup taxa

**Figure 4:**
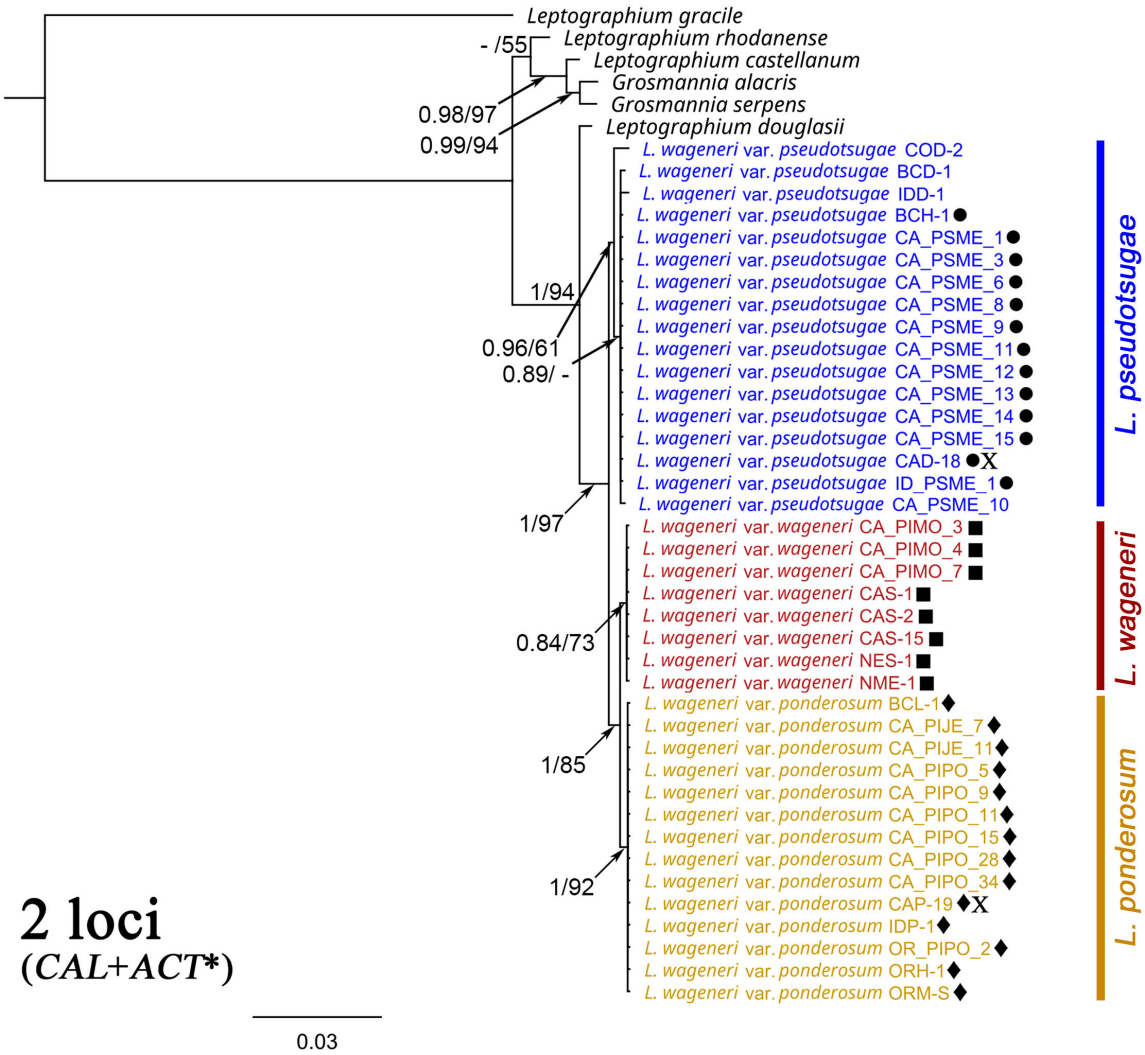
Phylogeny trees of the combined calmodulin (*CAL*) and actin (ACT*) loci with *Leptographium douglasii, L. rhodanense, L. gracile, L. castellanum, Grosmannia alacris*, and *G. serpens* [GenBank accession numbers (*CAL*/ACT*), KY424522/KY424502, KY424527/KY424506, MG205782/KM491324, JN135299/JN135324, JN135296/JN135318, and JN135300/JN135325, respectively] used as outgroup taxa. *Leptographium wageneri* (formerly *L*. *wageneri* var. *wageneri*), *L*. *ponderosum* (formerly *L. wageneri* var. *ponderosum*), and *L. pseudotsugae* (formerly *L. wageneri* var. *pseudotsugae*) are color-coded in red, yellow, and blue, respectively. The round, square, and diamond shapes inserted to the right of the isolate name indicate identical sequences. Numbers at each node indicate posterior probabilities (PP) greater than 0.80 and bootstrap support (BS) values greater than 50% (PP/BS). ACT* indicates actin dataset using Lepact-F & Lepact-R primers. Letter X indicates ex-type cultures of each variety.

For two loci, sequences were publicly available for six related *Leptographium* and *Grosmannia* species. A phylogenetic tree was generated based on concatenated sequences of *CAL* and ACT* of the *L. wageneri* s.l. isolates with those of *L. douglasii, L. rhodanense, L. gracile, L. castellanum, G.* alacris, and *G. serpens* (**Figure 4**). In this tree, each *L. wageneri* variety was well-supported: *L. wageneri* var. *wageneri* with 0.84 PP and 73 BS, *L. wageneri* var. *ponderosum* with 1 PP and 92 BS, and *L. wageneri* var. *pseudotsugae* with 0.96 PP and 61 BS.

### Taxonomy

Clear phylogenetic distinction as well as several minor morphological and physiological characters (Harrington and Cobb, 1986; Harrington and Cobb, 1987) (**Table 2**) provide strong justification for separating these three host-specialized, *Leptographium* pathogens as species. The low level of genetic diversity in the *L. wageneri* complex suggests that the three varieties represent three predominantly asexual lineages. The connection between *Leptographium wageneri* var. *ponderosum* and perithecia of *Ceratocystis wageneri* Goheen & F.W. Cobb [= *Ophiostoma wageneri* (Goheen & F.W. Cobb) T.C. Harr., = *Grosmannia wageneri* (Goheen & F.W. Cobb) Zipfel, Z.W. de Beer & M.J. Wingf., (MB#500831)] was not firmly established and was likely in error (Harrington, 1988). If not the teleomorph of *L. wageneri*, the ascospore shape and lack of a thick outer wall would place the purported teleomorph in *Grosmannia* (de Beer et al., 2022). Discounting the connection between *G. wageneri* and *L. wageneri*, the following three *Leptographium* species are proposed as the causes of black strain root disease on pinyons, hard pines, and Douglas-fir, respectively.

**Table 2:** The key ecological (host specificity), morphological, and physiological differences of three species: *Leptographium wageneri* (formerly *L*. *wageneri* var. *wageneri*), *L. ponderosum* (formerly *L. wageneri* var. *ponderosum*), and *L. pseudotsugae* (formerly *L. wageneri* var. *pseudotsugae*).

	*Leptographium wageneri* (formerly *L*. *wageneri* var. *wageneri*)	*L. ponderosum* (formerly *L. wageneri* var. *ponderosum*)	*L. pseudotsugae* (formerly *L. wageneri* var. *pseudotsugae*)	Reference
Primary hosts	Single-leaf pinyon (*Pinus monophyla*) and pinyon pine (*P. edulis*)	Ponderosa (*P. ponderosa*), Jeffrey (*P. jeffreyi*), and lodgepole (*P. contorta*) pines	Douglas-fir (*Pseudotsuga menziesii*)	From Harrington and Cobb, 1987
Mycelial pigment on PDA^a^	Yellowish-olive to hellebore green	Sudan brown to dresden brown	Yellowish-olive to hellebore green	From Harrington and Cobb, 1987
Conidiophore production on water agar^b^	Moderate to abundant	Sparse to none	Moderate to abundant	From Harrington and Cobb, 1987
Swelling of conidiophore at stipe apex^c^	0-2 μm	0-2 μm	2-6 μm	From Harrington and Cobb, 1987
Mean colony growth diameter^d^	35-42 mm Peak at 18 °C	35-50 mm Peak at 21 °C	48-55 mm Peak at 21 °C	From Harrington and Cobb, 1986

a After 7 days growth on potato dextrose agar (PDA) at 18°C. Colors are from Ridgway (1912).

b On colonized PDA plugs transferred to water agar and incubated for 10 days at 18°C.

c Width of stipe apex minus the width of the middle of the uppermost stipe cell. Range of means of 15 isolates, five conidiospores measures per isolate.

d After 7 days growth on PDA.

***Leptographium wageneri*** (W.B. Kendr.) M.J. Wingf., Trans. Brit. Mycol. Soc. 85 (1): 92 (1985) [MB#105458].

*Leptographium wageneri* var. *wageneri* (?) [MB#418941].

***Leptographium ponderosum*** (T.C. Harr. & F.W. Cobb) D. Choi, M.-S. Kim & T.C. Harr., comb. nov. [MB#849475].

*Verticicladiella wageneri* var. *ponderosa* T.C. Harr. & F.W. Cobb, Mycologia 78 (4): 566 (1986) [MB#117426].

*Leptographium wageneri* var. *ponderosum* (T.C. Harr. & F.W. Cobb) T.C. Harr. & F.W. Cobb, Mycotaxon 30: 505 (1987) [MB#133361].

*Holotype*: California, Siskyou County, McCloud Flat, from *Pinus ponderosa*, collected by T. C. Harrington, July 1980, UCB 1475041, ex-type isolate ATCC 58575 (= CAP-19).

***Leptographium pseudotsugae*** (T.C. Harr. & F.W. Cobb) D. Choi, M.-S. Kim & T.C. Harr., comb. nov. [MB#849460].

*Leptographium wageneri* var. *pseudotsugae* T.C. Harr. & F.W. Cobb, Mycotaxon 30: 505 (1987) [MB#133362].

*Holotype*: California, El Dorado County, Union Valley Res., from *Pseudotsuga menziesii*, collected by T. C. Harrington, July 1980, UCB 1475052, ex-type isolate ATCC 64196 (= CAD-18).

## Discussion

Because morphological features were once the predominate method available for classifying fungi (Hawksworth et al., 1996), early classification of the three *L. wageneri* varieties was based on their phenotypic characteristics, such as host-specialization, pigmentation of mycelia and conidiophore features (Harrington and Cobb, 1986; Harrington and Cobb, 1987) (**Table 2**). Subsequently, isozyme variation and RAPD markers supported distinction of the three varieties (Otrosina and Cobb, 1987; Zambino and Harrington, 1989; Zambino and Harrington, 1992; Witthuhn et al., 1997). Phylogenetic analyses based on rDNA (ITS2 and LSU) sequences did not provide clear separation of the three *L. wageneri* varieties (Jacobs et al., 2001). The present study is the first to demonstrate robust separation of the three *L. wageneri* varieties using multiple, non-rDNA loci. Although Bennett et al. (2021) demonstrated strong separation between two varieties (*L. wageneri* var. *wageneri* and *L. wageneri* var. *pseudotsugae*) based on over 100,000 binary SNPs, their analyses included isolates from limited geographic areas (e.g., *L. wageneri* var. *pseudotsugae* isolates from Oregon and *L. wageneri* var. *wageneri* isolates from California). And, sequences of the three most parsimony informative loci (e.g., *CAL*, ACT*, and *TEF-1α*) from *L. wageneri* var. *pseudotsugae* isolates were identical to those within the genomic sequences of *L. wageneri* var. *pseudotsugae* isolates from Oregon used in the study of Bennett et al. (2021). The clear phylogenetic separation warrants species designation.

Phylogenetic separation of *L. wageneri*, *L. ponderosum* and *L. pseudotsugae* was better resolved when multi-locus analyses were used rather than a single locus. In general, these three *Leptographium* species showed very little genetic variation, and most of the loci showed only minor sequence differences. When analyzing with a single locus, these three species tended to be separated differently depending on the locus being analyzed. For example, for *MAT1-1-3* and *TEF-1α*, *L. wageneri* was distinct, while for ACT** (ACT512F & ACT783R) and *GPD*, *L. ponderosum* was distinct, and for *CHS* and *CAL*, *L. pseudotsugae* was distinct. The very limited genetic variation within each of these three *Leptographium* species, pathogens generally believed to be native to western North America, strongly suggests that they are predominantly asexual.

In most cases, ACT* (Lepact-F & Lepact-R) could be used to identify the three *Leptographium* species. ACT*-based separation of three species was generally well-supported, with the exception of one isolate, IDD-1 (*L. pseudotsugae*), which was placed in the *L. wageneri* clade. The *TUB* locus has been used for identification at the species level; however, *TUB*-based identification of *L. ponderosum* is distinguished by only a single-base substitution.

Because the majority of parsimony-informative sites were found in the *CAL*, ACT* (Lepact-F & Lepact-R), and *TEF-1α* loci, combinations among these loci were found to be most useful in separating the *Leptographium* species. Of these three loci, *CAL* had the most parsimony-informative sites. However, with the *CAL*-based tree, only *L. pseudotsugae* was separated. A phylogeny based on *CAL* with ACT* (Lepact-F & Lepact-R), which had the next most parsimony-informative sites, resulted in divergence and separation of three species. In the phylogenetic analysis of three combined-loci, *CAL*, ACT* (Lepact-F & Lepact-R), and *TEF-1α*, the separation of all three *Leptographium* species was clear and strongly supported. These results suggest that cumulative phylogenetic information provides more precise divergence with stronger support values for examining evolutionary relationships within and among the species (Taylor et al., 2000).

Distinguishing different species of closely related fungi is a complex process that considers various factors including morphology, biological reproduction, biochemical properties, ecological behaviors, genetic differences, and other factors (Mayr, 1940; Hennig, 1965; Hawksworth et al., 1996; Harrington and Rizzo, 1999; Taylor et al., 2000). In the case of *L. wageneri*, the three host-specialized pathogens (Harrington and Cobb, 1984) were previously described as varieties instead of species, largely because phylogenetic data were lacking to distinguish these taxa. However, differences among the three *L. wageneri* varieties were previously noted in terms of minor morphological characteristics, isozyme variations, RAPD profiles, and ecological habitat (host species) (Harrington and Cobb, 1986; Harrington and Cobb, 1987; Otrosina and Cobb, 1987; Zambino and Harrington, 1989; Zambino and Harrington, 1992; Witthuhn et al., 1997). When recognizing new species within a genus, multiple genes have been used in phylogenetic analyses to allow for concordance (Taylor et al., 2000), and several new *Leptographium* species have been described using multi-locus datasets (Kim et al., 2004; Lee et al., 2005; Jacobs et al., 2006). In this study, the three varieties of *L. wageneri* were clearly separated phylogenetically on the basis of concatenated sequences of 10 loci and on the two-loci dataset with multiple outgroup taxa. Thus, solid genetic evidence is provided that the three *L. wageneri* varieties should be elevated to species status. Recognition of the *L. wageneri* varieties as three separate *Leptographium* species will help to understand ecological interactions and evolutionary history of these forest pathogens, which can contribute to improved management of the black stain root disease pathosystem.

## Data availability statement

The datasets presented in this study can be found in online repositories. The names of the repository/repositories and accession number(s) can be found in the article/**Supplementary Material**.

## Author contributions

DC: Data curation, Formal analysis, Methodology, Writing – original draft, Software, Visualization. TH: Data curation, Methodology, Investigation, Validation, Writing – review & editing. DS: Investigation, Writing – review & editing, Resources, Supervision. JS: Conceptualization, Data curation, Formal analysis, Funding acquisition, Methodology, Validation, Writing – review & editing. DK: Writing – review & editing, Data curation, Formal analysis, Methodology, Software. NK: Writing – review & editing, Conceptualization, Funding acquisition, Investigation, Resources. M-SK: Conceptualization, Funding acquisition, Investigation, Resources, Writing – review & editing, Data curation, Formal analysis, Methodology, Project administration, Supervision, Validation.
