# Supplementary material for: Phylogenetic analyses allow species-level recognition of Leptographium wageneri varieties that cause black stain root disease of conifers in western North America
Source: Front Plant Sci. 2023 Dec 22;14:1286157. doi: 10.3389/fpls.2023.1286157 (PMC10777841; doi:10.3389/fpls.2023.1286157)
Supplement: Supplementary file 1 [file DataSheet_1.pdf]

Supplementary Material

1 Supplementary Tables and Figures

1.1 Supplementary Tables

Supplemental Table 1. Host species, DBH (diameter at breast height), GPS points and/or general locations for 8 *Leptographium wageneri* (formerly *L. wageneri* var. *wageneri*), 14 *L. ponderosum* (formerly *L. wageneri* var. *ponderosum*), and 17 *L. pseudotsugae* (formerly *L. wageneri* var. *pseudotsugae*) isolates.

Sample ID (Live/Dead)	Host	DBH (cm)	GPS	General Location	Collection Date (mm/dd/yyyy or yyyy)	Collector
			Latitude, Longitude			
Leptographium wageneri (formerly *L. wageneri* var. *wageneri*)						
CA_PIMO_3 (L)	*Pinus monophylla*	16.5	37°21.114 N, 118°10.946 W	White Mountain, CA, USA	07/12/2021	Daram Choi[1], Sukhyun Joo[1], David C. Shaw[1], Martin Mackenzie[2]
CA_PIMO_4 (L)	*Pinus monophylla*	9.9	37°21.115 N, 118°10.942 W	White Mountain, CA, USA	07/12/2021	Daram Choi[1], Sukhyun Joo[1], David C. Shaw[1], Martin Mackenzie[2]
CA_PIMO_7 (L)	*Pinus monophylla*	4.1	37°21.267 N, 118°10.890 W	White Mountain, CA, USA	07/12/2021	Daram Choi[1], Sukhyun Joo[1], David C. Shaw[1], Martin Mackenzie[2]
CAS-1 (-)	*Pinus monophylla*	-	-	San Bernardino, CA, USA	04/23/1980	Thomas C. Harrington[3]
CAS-2 (-)	*Pinus monophylla*	-	-	San Bernardino, CA, USA	04/23/1980	Thomas C. Harrington[3]
CAS-15 (-)	*Pinus monophylla*	-	-	White Mountain, CA, USA	08/15/1981	Thomas C. Harrington[3]
NES-1 (-)	*Pinus monophylla*	-	-	NE, USA	08/14/1981	Thomas C. Harrington[3]
NME-1 (-)	*Pinus edulis*	-	-	NM, USA	1976	Thomas C. Harrington[3]
Leptographium ponderosum (formerly *L. wageneri* var. *ponderosum*)						
OR_PIPO_2 (L)	*Pinus ponderosa*	21.7	44°23.005 N, 121°33.176 W	Sisters, OR, USA	03/26/2021	Daram Choi[1], David C. Shaw[1], Brent W. Oblinger[4]
CA_PIPO_9 (L)	*Pinus ponderosa*	50.4	40°11.563 N, 121°10.798 W	Pratt, CA, USA	07/15/2021	Daram Choi[1], Sukhyun Joo[1], David C. Shaw[1], William Woodruff[5], Ashley E. Hawkins[6], Mee-Sook Kim[7]
CA_PIPO_11 (L)	*Pinus ponderosa*	54	40°12.009 N, 121°10.552 W	Pratt, CA, USA	07/15/2021	Daram Choi[1], Sukhyun Joo[1], David C. Shaw[1], William Woodruff[5], Ashley E. Hawkins[6], Mee-Sook Kim[7]

CA_PIPO_15 (L)	*Pinus ponderosa*	13.9	40°42.650 N, 121°09.797 W	Black Mountain Experimental Forest, CA, USA	07/15/2021	Daram Choi[1], Sukhyun Joo[1], David C. Shaw[1], William Woodruff[5], Ashley E. Hawkins[6], Mee-Sook Kim[7]
CA_PIPO_28 (L)	*Pinus ponderosa*	27.3	41°59.328 N, 120°48.413 W	Crowder Flat2, CA, USA	07/16/2021	Daram Choi[1], Sukhyun Joo[1], David C. Shaw[1], William Woodruff[5], Ashley E. Hawkins[6], Mee-Sook Kim[7]
CA_PIPO_34 (L)	*Pinus ponderosa*	Height below breast height	40°11.557 N, 121°10.825 W	Pratt, CA, USA	07/15/2021	Daram Choi[1], Sukhyun Joo[1], David C. Shaw[1], William Woodruff[5], Ashley E. Hawkins[6], Mee-Sook Kim[7]
CA_PIJE_7 (L)	*Pinus jeffreyi*	28.8	41°00.374 N, 120°45.614 W	Heart Rock, CA, USA	07/15/2021	Daram Choi[1], Sukhyun Joo[1], David C. Shaw[1], William Woodruff[5], Ashley E. Hawkins[6], Mee-Sook Kim[7]
CA_PIJE_11 (L)	*Pinus jeffreyi*	17	41°00.376 N, 120°45.639 W	Heart Rock, CA, USA	07/15/2021	Daram Choi[1], Sukhyun Joo[1], David C. Shaw[1], William Woodruff[5], Ashley E. Hawkins[6], Mee-Sook Kim[7]
BCL-1 (-)	*Pinus contorta*	-	-	Monashee, BC, Canada	03/17/1980	Thomas C. Harrington[3]
ORH-1 (-)	*Tsuga heterophylla*	-	-	Mt. Hood, OR, USA	Unknown	Thomas C. Harrington[3]
ORM-S (-)	*Tsuga mertensiana*	-	-	Warm Springs, OR, USA	Unknown	Thomas C. Harrington[3]
IDP-1 (-)	*Pinus ponderosa*	-	-	City of Rocks, ID, USA	1980	Thomas C. Harrington[3]
CAP-19 (-)	*Pinus ponderosa*	-	-	McCloud, CA, USA	07/27/1980	Thomas C. Harrington[3]
CA_PIPO_5 (-)	*Pinus ponderosa*	-	-	Roney, CA, USA	08/29/2019	William Woodruff[5], Ned B. Klopfenstein[8], Emmanuel Duarte-Mata[9], Mee-Sook Kim[7]
Leptographium pseudotsugae (formerly *L. wageneri* var. *pseudotsugae*)						
CA_PSME_6 (L)	*Pseudotsuga menziesii*	22.8	40°27.570 N, 123°36.930 W	Sulphur Valley, CA, USA	07/07/2021	Daram Choi[1], Sukhyun Joo[1], David C. Shaw[1], Christopher A. Lee[10], Ashley E. Hawkins[6]
CA_PSME_8 (L)	*Pseudotsuga menziesii*	11.7	40°27.377 N, 123°36.597 W	Sulphur Valley, CA, USA	07/07/2021	Daram Choi[1], Sukhyun Joo[1], David C. Shaw[1], Christopher A. Lee[10], Ashley E. Hawkins[6]
CA_PSME_9 (L)	*Pseudotsuga menziesii*	7.9	40°27.475 N, 123°36.579 W	Sulphur Valley, CA, USA	07/07/2021	Daram Choi[1], Sukhyun Joo[1], David C. Shaw[1], Christopher A. Lee[10], Ashley E. Hawkins[6]
CA_PSME_10 (L)	*Pseudotsuga menziesii*	24.1	40°27.543 N, 123°36.571 W	Sulphur Valley, CA, USA	07/07/2021	Daram Choi[1], Sukhyun Joo[1], David C. Shaw[1], Christopher A. Lee[10], Ashley E. Hawkins[6]
CA_PSME_11 (L)	*Pseudotsuga menziesii*	33.2	39°21.917 N, 123°32.479 W	Jackson State Forest, CA, USA	07/08/2021	Daram Choi[1], Sukhyun Joo[1], David C. Shaw[1], Christopher A. Lee[10], Ashley E. Hawkins[6]
CA_PSME_12 (D)	*Pseudotsuga menziesii*	5.6	39°20.773 N, 123°30.565 W	Jackson State Forest, CA, USA	07/08/2021	Daram Choi[1], Sukhyun Joo[1], David C. Shaw[1], Christopher A. Lee[10], Ashley E. Hawkins[6]
CA_PSME_13 (L)	*Pseudotsuga menziesii*	17.2	39°21.586 N, 123°39.176 W	Jackson State Forest, CA, USA	07/08/2021	Daram Choi[1], Sukhyun Joo[1], David C. Shaw[1], Christopher A. Lee[10], Ashley E. Hawkins[6]
CA_PSME_14 (L)	*Pseudotsuga menziesii*	27.4	39°21.614 N, 123°39.237 W	Jackson State Forest, CA, USA	07/08/2021	Daram Choi[1], Sukhyun Joo[1], David C. Shaw[1], Christopher A. Lee[10], Ashley E. Hawkins[6]
CA_PSME_15 (D)	*Pseudotsuga menziesii*	17.6	39°22.032 N, 123°39.040 W	Jackson State Forest, CA, USA	07/08/2021	Daram Choi[1], Sukhyun Joo[1], David C. Shaw[1], Christopher A. Lee[10], Ashley E. Hawkins[6]
BCH-1 (-)	*Tsuga heterophylla*	-	-	Tugwell, BC, Canada	03/17/1980	Thomas C. Harrington[3]

BCD-1 (-)	*Pseudotsuga menziesii*	-	-	Cicero, BC, Canada	03/17/1980	Thomas C. Harrington[3]
CAD-18 (-)	*Pseudotsuga menziesii*	-	-	Spring Valley, CA, USA	07/27/1980	Thomas C. Harrington[3]
COD-2 (-)	*Pseudotsuga menziesii*	-	-	Evergreen, CO, USA	1981	Thomas C. Harrington[3]
IDD-1 (-)	*Pseudotsuga menziesii*	-	-	Jerry Johnson campground, ID, USA	06/20/1980	Thomas C. Harrington[3]
ID_PSME_1 (-)	*Pseudotsuga menziesii*	-	-	Farragut State Park, ID, USA	11/05/2019	Paul J. Zambino[8]
CA_PSME_1 (-)	*Pseudotsuga menziesii*	-	-	Tuolumne County, CA, USA	2019	Martin MacKenzie[2]
CA_PSME_3 (-)	*Pseudotsuga menziesii*	-	-	Friday Ridge Rd, CA, USA	10/31/2019	Christopher A. Lee[10]

[1]Oregon State University, Department of Forest Engineering, Resources & Management, Corvallis, OR, United States, [2]USDA Forest Service, South Sierra Shared Service Area, Sonora, CA, United States, [3]Iowa State University, Department of Plant Pathology, Entomology and Microbiology, Ames, IA, United States, [4]USDA Forest Service, Pacific Northwest Region, Bend, OR, United States, [5]USDA Forest Service, Northeastern California Shared Service Area, Susanville, CA, United States, [6]USDA Forest Service, Northern California Shared Service Area, Redding, CA, United States, [7]USDA Forest Service, Pacific Northwest Research Station, Corvallis, OR, United States, [8]USDA Forest Service, Rocky Mountain Research Station, Moscow, ID, United States, [9]University of Guanajuato, Division of Life Sciences, Department of Agronomy, Irapuato, Guanajuato, Mexico, [10]California Department of Forestry and Fire Protection, North Coast, Fortuna, CA, United States.

Supplemental Table 2. Primers with direction, sequences, and references used for phylogenetic analyses.

Locus	Primer	Direction	Sequence (5′-3′)	Reference
28S large subunit rDNA (LSU)	LR0R<br>LR5	Forward<br>Reverse	ACC CGC TGA ACT TAA GC<br>TCC TGA GGG AAA CTT CG	Rehner and Samuels, 1994<br>Vilgalys and Hester, 1990
Actin (*ACT**)	Lepact-F<br>Lepact-R	Forward<br>Reverse	TAC GTC GGT GAC GAG GC<br>CAA TGA TCT TGA CCT TCA T	Lim et al., 2004
Actin (*ACT***)	ACT512F<br>ACT783R	Forward<br>Reverse	ATG TGC AAG GCC GGT TTC GC<br>TAC GAG TCC TTC TGG CCC AT	Carbone and Kohn, 1999
β-tubulin (*TUB*)	Bt2a<br>Bt2b	Forward<br>Reverse	GGT AAC CAA ATC GGT GCT GCT TTC<br>ACC CTC AGT GTA GTG ACC CTT GGC	Zipfel et al., 2005
Calmodulin (*CAL*)	CL2F<br>CL2R	Forward<br>Reverse	GAC AAG GAY GGY GAT GGT<br>TTC TGC ATC ATG AGY TGS AC	Duong et al., 2012
Translation elongation factor 1-alpha (*TEF-1α*)	EF1-F<br>EF2-R	Forward<br>Reverse	TGC GGT GGT ATC GAC AAG CGT<br>AGC ATG TTG TCG CCG TTG AAG	Jacobs et al., 2004
Mating-type gene (*MAT1-1-3*)	Oph-MAT1F1<br>Oph-MAT1R2	Forward<br>Reverse	ATG KCC RAT GAR GAY TGC T<br>GGC GKT KGC RTT GTA YTT GTA	Duong et al., 2016
RNA polymerase II subunit (*RPB2*)	fRPB2-5F<br>fRPB2-7cR	Forward<br>Reverse	GAY GAY MGW GAT CAY TTY GG<br>CCC ATR GCT TGY TTR CCC AT	Liu et al., 1999
Glyceraldehyde-3-phosphate dehydrogenase (*GPD*)	GPD10F<br>GPD522R	Forward<br>Reverse	GCN TCN TGC ACV ACS AAC TG<br>YCC SRA CTC GTT GTC GTA CC	Antonín et al., 2021
Chitin synthase (*CHS*)	CHS-79F<br>CHS-354R	Forward<br>Reverse	TGG GGC AAG GAT GCT TGG AAG AAG<br>TGG AAG AAC CAT CTG TGA GAG TTG	Carbone and Kohn, 1999

Supplemental Table 3. Denaturation, annealing, and extension temperature parameters of thermocycler settings for each primer set used in this study.

Locus[1]<br>Primer Set	PCR Setting		
	Initial Denaturation	Repeat Cycle	Final Extension
LSU<br>LR0R and LR5	95 °C 3 min	35 cycles of 95 °C for 30 s, annealing at 55 °C for 30 s, and 72 °C for 1min	72 °C 10 min
*ACT**<br>Lepact-F and Lepact-R	94 °C 4 min	30 cycles of 94 °C for 30 s, annealing at 55 °C for 50 s, and 72 °C for 50 s	72 °C 10 min
*ACT***<br>ACT512F and ACT783R	95 °C 8 min	35 cycles of 95 °C for 15 s, annealing at 55 °C for 20 s, and 72 °C for 1min	72 °C 5 min
TUB<br>Bt2a and Bt2b	94 °C 3 min	30 cycles of 94 °C for 30 s, annealing at 55 °C for 50 s, and 72 °C for 50 s	72 °C 10 min
CAL<br>CL2F and CL2R	95 °C 5 min	35 cycles of 95 °C for 30 s, annealing at 55 °C for 30 s, and 72 °C for 1min	72 °C 8 min
TEF-1α<br>EF1-F and EF2-R	95 °C 3 min	30 cycles of 95 °C for 30 s, annealing at 55 °C for 45 s, and 72 °C for 1min	72 °C 8 min
MAT1-1-3<br>Oph-MAT1F1 and Oph-MAT1R2	95 °C 5 min	35 cycles of 95 °C for 30 s, annealing at 55 °C for 30 s, and 72 °C for 1min	72 °C 8 min
RPB2<br>fRPB2-5F and fRPB2-7cR	94 °C 1 min	35 cycles of 95 °C for 30 s, annealing at 56 °C for 30 s, and 72 °C for 45 s	72 °C 10 min
GPD<br>GPD10F and GPD522R	94 °C 1 min	35 cycles of 95 °C for 30 s, annealing at 55 °C for 30 s, and 72 °C for 4 5s	72 °C 10 min
CHS<br>CHS-79F and CHS-354R	95 °C 6 min	30 cycles of 94 °C for 1 min, annealing at 55 °C for 2 min, and 72 °C for 3min	72 °C 10 min

[1]Loci are described in Supplemental Table 2.

Supplemental Table 4. Locus name, sequence length of individual and combined genes, parsimony-informative sites of individual genes, outgroup used for the locus/loci, and the GenBank accession numbers used in the phylogenetic analyses.

Number of Locus	Locus[1]	Length (bp)	Parsimony-Informative Site	Outgroup	Accession No.
1 Locus	LSU	804	0	*Leptographium douglasii*	OM514766
	*ACT** (Lepact-F & Lepact-R)	816	4	*Leptographium douglasii*	KY424502
	*ACT*** (ACT512F & ACT783R)	288	1	*Leptographium douglasii*	-
	TUB	337	0	*Leptographium douglasii*	KY424512
	CAL	693	9	*Leptographium douglasii*	KY424522
	TEF-1α	565	2	*Leptographium douglasii*	KY424532
	MAT1-1-3	446	1	*Leptographium douglasii*	KT779230
	RPB2	1,059	0	*Leptographium douglasii*	OM631627
	GPD	544	1	*Leptographium douglasii*	-
	CHS	215	1	*Leptographium douglasii*	-
2 Loci	*CAL*<br>*ACT** (Lepact-F & Lepact-R)	1,494	-	*Leptographium douglasii*	
3 Loci	*CAL*<br>*ACT** (Lepact-F & Lepact-R)<br>*TEF-1α*	2,059	-	*Leptographium douglasii*	
10 Loci	LSU, *ACT** (Lepact-F & Lepact-R), *ACT*** (ACT512F & ACT783R), *TUB*, *CAL*, *TEF-1α*, *MAT1-1-3*, *RPB2*, *GPD*, and *CHS*	5,767	-	*Leptographium douglasii*	-
2 Loci	*CAL*<br>*ACT** (Lepact-F & Lepact-R)	1,531	-	*Leptographium douglasii*	KY424522<br>KY424502
				Leptographium rhodanense	KY424527<br>KY424506
				Leptographium gracile	MG205782<br>KM491324
				Leptographium castellanum	JN135299<br>JN135324
				Grosmannia alacris	JN135296<br>JN135318
				Grosmannia serpens	JN135300<br>JN135325

[1]Loci are described in Supplemental Table 2.

Supplemental Table 5. Evolutionary substitution models used for maximum likelihood (ML) and Bayesian analyses.

Number of Loci	Tree	ML Model	Reference	Bayesian Model	Reference
1 Locus	LSU[1]	F81+F	Felsenstein (1981)	F81	Felsenstein (1981)
	*ACT** (Lepact-F & Lepact-R)	HKY+F	Hasegawa et al. (1985)	HKY	Hasegawa et al. (1985)
	*ACT*** (ACT512F & ACT783R)	F81+F	Felsenstein (1981)	F81	Felsenstein (1981)
	TUB	JC	Jukes and Cantor (1969)	JC	Jukes and Cantor (1969)
	CAL	K2P	Kimura (1980)	K2P	Kimura (1980)
	TEF-1α	K2P	Kimura (1980)	K2P	Kimura (1980)
	MAT1-1-3	K2P	Kimura (1980)	K2P	Kimura (1980)
	RPB2	TN+F	Tamura and Nei (1993)	HKY	Hasegawa et al. (1985)
	GPD	F81+F	Felsenstein (1981)	F81	Felsenstein (1981)
	CHS	JC	Jukes and Cantor (1969)	JC	Jukes and Cantor (1969)
2 Loci	*CAL* + *ACT** (Lepact-F & Lepact-R)	HKY+F+I	Hasegawa et al. (1985)	HKY+I	Hasegawa et al. (1985)
3 Loci	*CAL* + *ACT** (Lepact-F & Lepact-R) + *TEF-1α*	TN+F+I	Tamura and Nei (1993)	HKY+I	Hasegawa et al. (1985)
10 Loci	LSU, *ACT** (Lepact-F & Lepact-R), *ACT*** (ACT512F & ACT783R), *TUB*, *CAL*, *TEF-1α*, *MAT1-1-3*, *RPB2*, *GPD*, and *CHS*	TN+F+I	Tamura and Nei (1993)	HKY+I	Hasegawa et al. (1985)
2 Loci	*CAL* + *ACT** (Lepact-F & Lepact-R) with six outgroups: *Leptographium douglasii, L. rhodanense, L. gracile, L. castellanum, Grosmannia. alacris,* and *G. serpens*	TN+F+I	Tamura and Nei (1993)	HKY+I	Hasegawa et al. (1985)

[1]Loci are described in Supplemental Table 2.

References

Antonín, V., Stewart, J.E., Ortiz, R.M., Kim, M.-S., Bonello, P., Tomšovský, M., Klopfenstein, N.B. 2021. *Desarmillaria caespitosa*, a North American vicariant of *D. tabescens*. Mycologia 113, 776-790.

Carbone, I., Kohn, L.M. 1999. A method for designing primer sets for speciation studies in filamentous ascomycetes. Mycologia 91, 553-556.

Duong, T.A., de Beer, Z.W., Wingfield, B.D., Wingfield, M.J. 2012. Phylogeny and taxonomy of species in the *Grosmannia serpens* complex. Mycologia 104, 715-732.

Duong, T.A., de Beer, Z.W., Wingfield, B.D., Wingfield, M.J. 2016. Mating type markers reveal high levels of heterothallism in *Leptographium sensu lato*. Fungal Biology 120, 538-546.

Felsenstein, J. 1981. Evolutionary trees from DNA sequences: a maximum likelihood approach. Journal of Molecular Evolution 17, 368-376.

Hasegawa, M., Kishino, H., Yano, T. 1985. Dating of the human-ape splitting by a molecular clock of mitochondrial DNA. Journal of Molecular Evolution 22, 160-174.

Jacobs, K., Bergdahl, D.R., Wingfield, M.J., Halik, S., Seifert, K.A., Bright, D.E., Wingfield, B.D. 2004. *Leptographium wingfieldii* introduced into North America and found associated with exotic *Tomicus piniperda* and native bark beetles. Mycological Research 108, 411-418.

Jukes, T.H., Cantor, C.R. 1969. Evolution of protein molecules. In: Munro, H.N., Ed., Mammalian Protein Metabolism, Academic Press, New York, 21-132.

Kimura, M. 1980. A simple method for estimating evolutionary rates of base substitutions through comparative studies of nucleotide sequences. Journal of Molecular Evolution 16, 111-120.

Lim, Y.W., Alamouti, S.M., Kim, J.-J., Lee, S., Breuil, C. 2004. Multigene phylogenies of *Ophiostoma clavigerum* and closely related species from bark beetle-attacked *Pinus* in North America. FEMS Microbiology Letters 237, 89-96.

Liu, Y.J., Whelen, S., Hall, B.D. 1999. Phylogenetic relationships among ascomycetes: evidence from an RNA polymerse II subunit. Molecular Biology and Evolution 16, 1799-1808.

Rehner, S.A., Samuels, G.J. 1994. Taxonomy and phylogeny of *Gliocladium* analysed from nuclear large subunit ribosomal DNA sequences. Mycological Research 98, 625-634.

Vilgalys, R., Hester, M. 1990. Rapid genetic identification and mapping of enzymatically amplified ribosomal DNA from several *Cryptococcus* species. Journal of bacteriology 172, 4238-4246.

Zipfel, R.D., De Beer, Z.W., Jacobs, K., Wingfield, B.D., Wingfield, M.J. 2005. Multi-gene phylogenies define *Ceratocystiopsis* and *Grosmannia* distinct from *Ophiostoma*. Studies in Mycology 55, 75-97.

1.2 Supplementary Figures

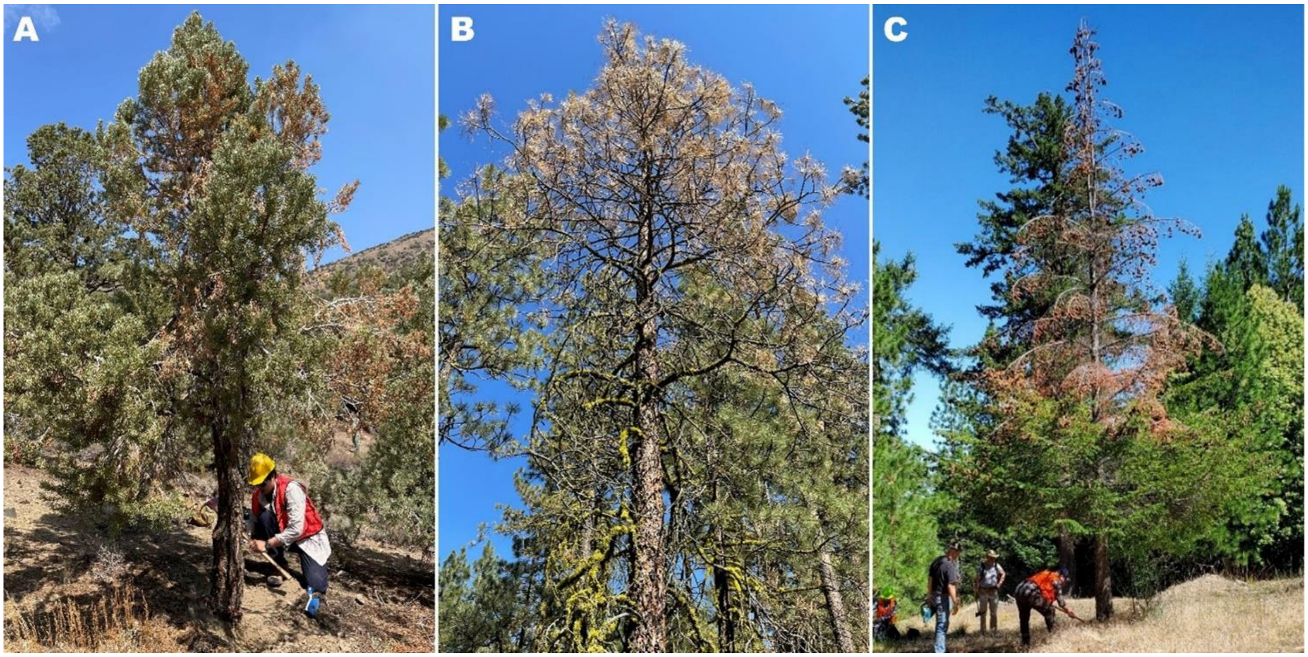

Supplemental Figure 1. *Leptographium wageneri*-infected trees show typical crown symptoms such as needle discoloration, needle loss, and tufted needles. Symptoms are showing on (A) a single-leaf pinyon pine (*Pinus monophylla*), (B) ponderosa pine (*Pinus ponderosa*), and (C) Douglas-fir (*Pseudotsuga menziesii*).

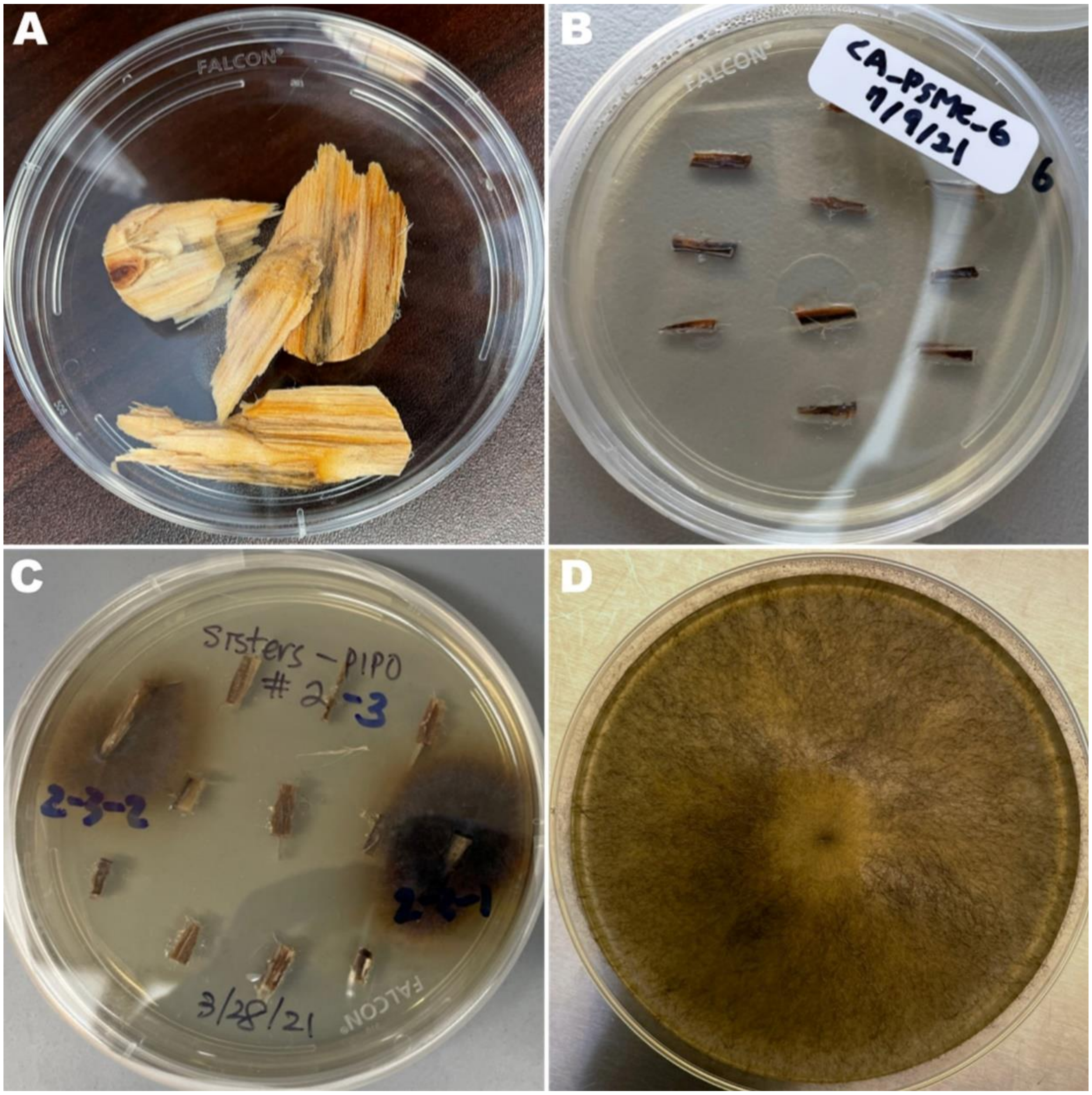

Supplemental Figure 2. Fungal isolation process for *Leptographium wageneri* s.l. from collected field samples. (A) Wood samples with stain, (B) embedded wooden pieces (ca. 10 x 2.5 x 2.5 mm) on a selective, malt-extract agar medium amended with cycloheximide and streptomycin sulfate, (C) the growth of dark brown to black colored hyphae, and (D) the growth of a hyphal tip-derived culture.

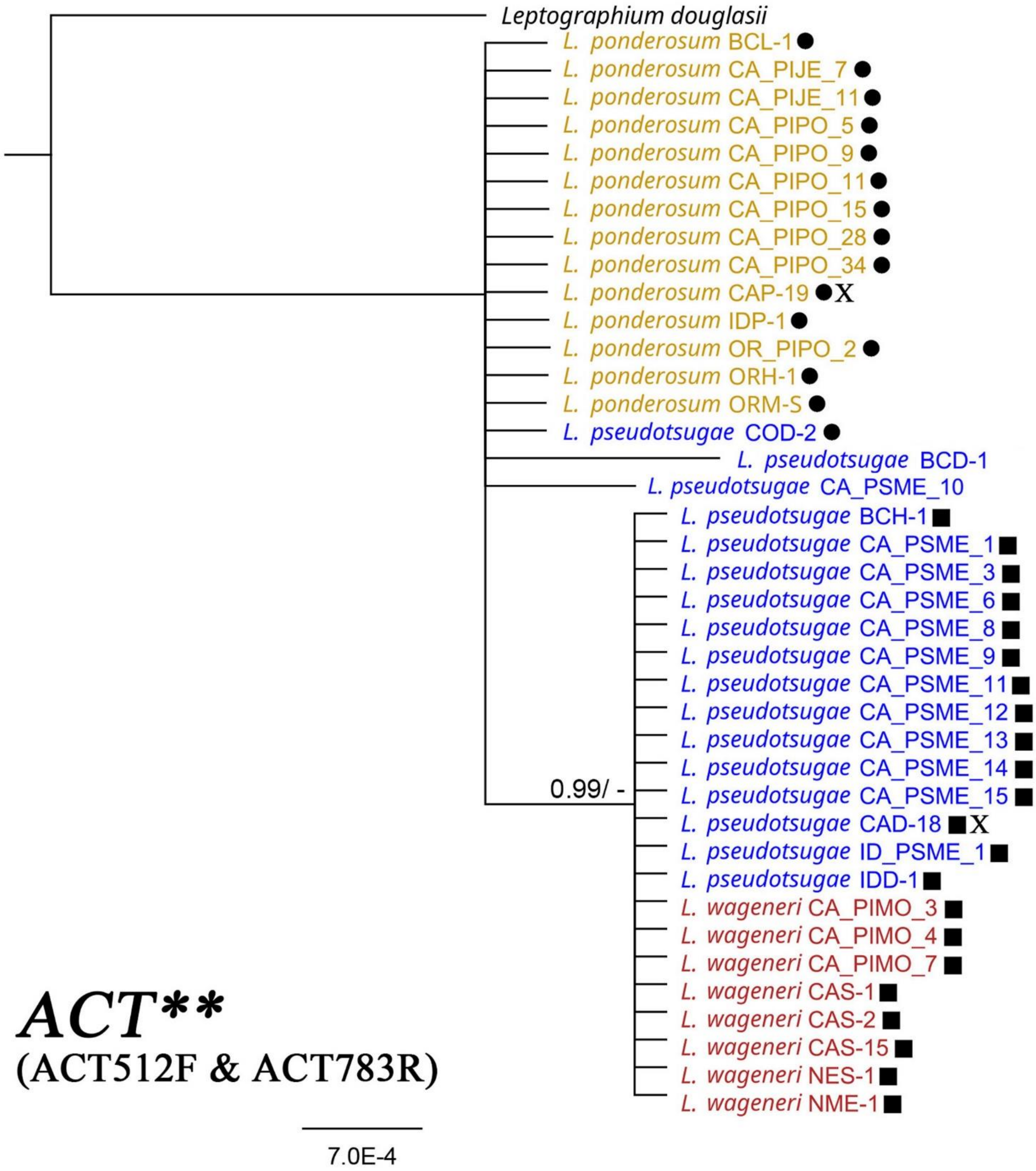

Supplemental Figure 3. Phylogeny of actin (*ACT***) locus with *Leptographium douglasii* used as the outgroup. *Leptographium wageneri* (formerly *L. wageneri* var. *wageneri*), *L. ponderosum* (formerly *L. wageneri* var. *ponderosum*), and *L. pseudotsugae* (formerly *L. wageneri* var. *pseudotsugae*) are color-coded in red, yellow, and blue, respectively. The round and square shapes inserted to the right of the isolate name indicate identical sequences. Numbers at each node indicate posterior probabilities (PP) greater than 0.80 and bootstrap support (BS) values greater than 50% (PP/BS). *ACT*** indicates actin dataset using ACT512F & ACT783R primers. Letter X indicates ex-type cultures of each variety.

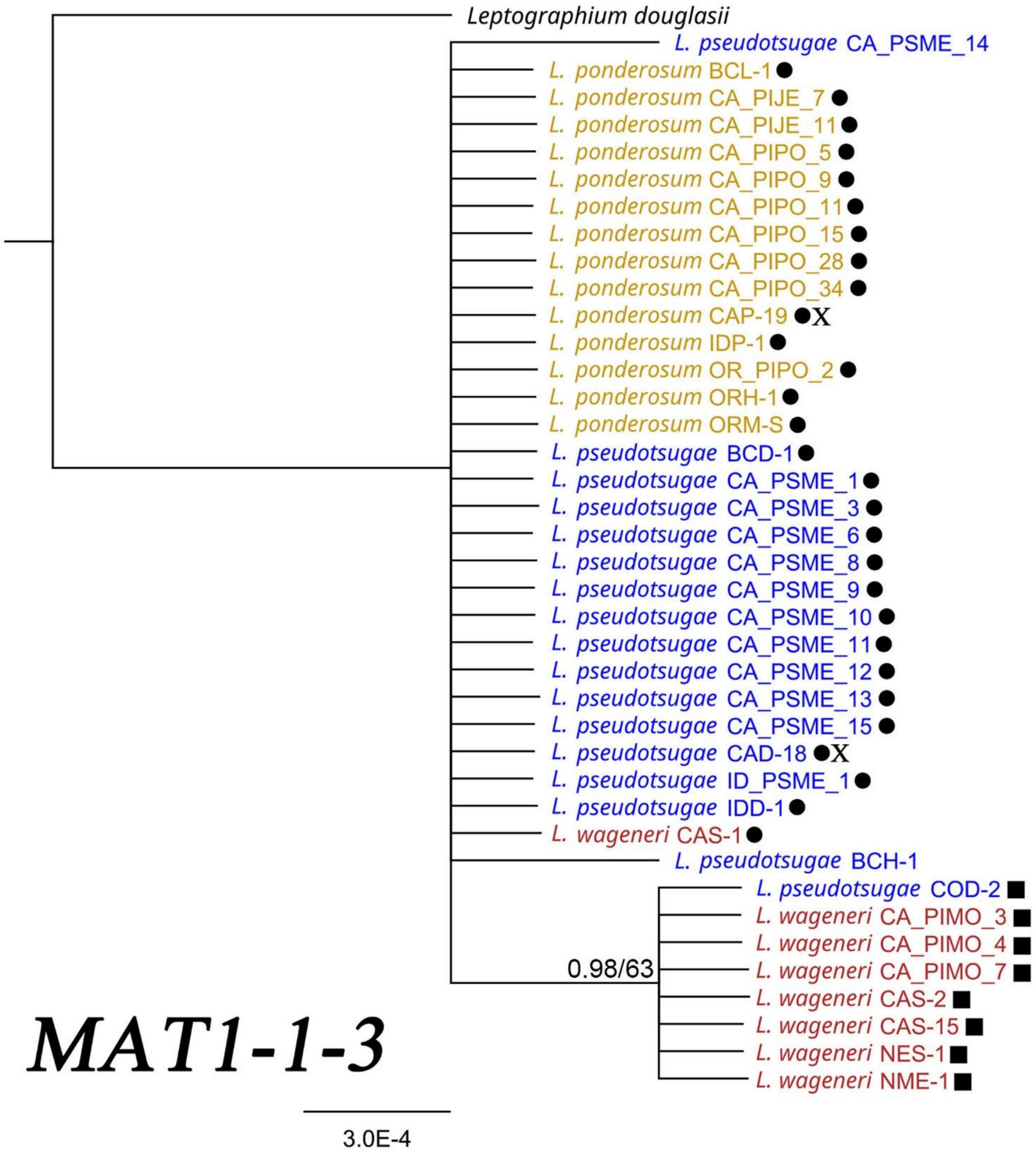

Supplemental Figure 4. Phylogeny of mating-type gene *MAT1-1-3* with *Leptographium douglasii* (GenBank accession #KT779230) used as the outgroup. *Leptographium wageneri* (formerly *L. wageneri* var. *wageneri*), *L. ponderosum* (formerly *L. wageneri* var. *ponderosum*), and *L. pseudotsugae* (formerly *L. wageneri* var. *pseudotsugae*) are color-coded in red, yellow, and blue, respectively. The round and square shapes inserted to the right of the isolate name indicate identical sequences. Numbers at each node indicate posterior probabilities (PP) greater than 0.80 and bootstrap support (BS) values greater than 50% (PP/BS). Letter X indicates ex-type cultures of each variety.

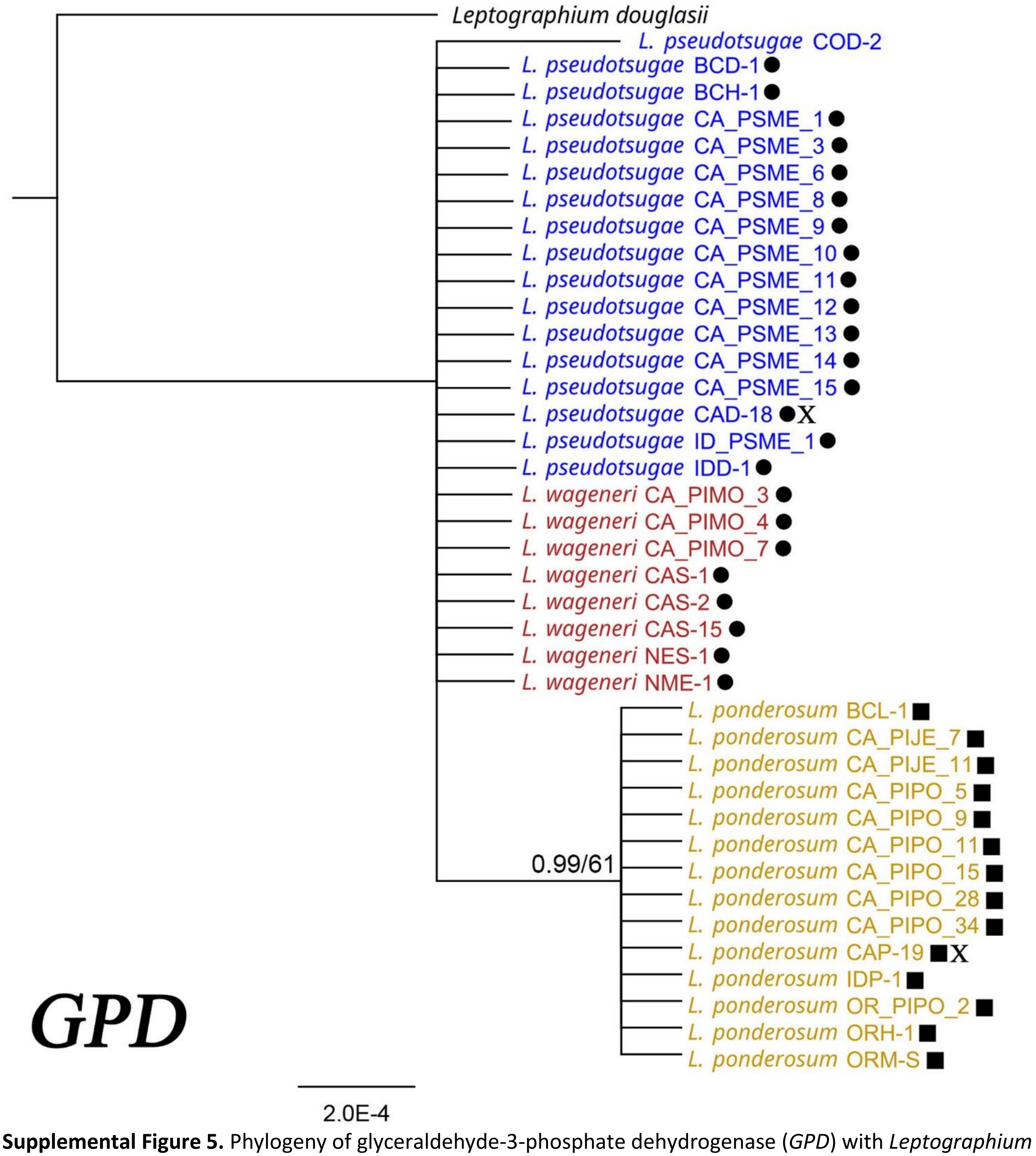

Supplemental Figure 5. Phylogeny of glyceraldehyde-3-phosphate dehydrogenase (*GPD*) with *Leptographium douglasii* used as the outgroup. *Leptographium wageneri* (formerly *L. wageneri* var. *wageneri*), *L. ponderosum* (formerly *L. wageneri* var. *ponderosum*), and *L. pseudotsugae* (formerly *L. wageneri* var. *pseudotsugae*) are color-coded in red, yellow, and blue, respectively. The round and square shapes inserted to the right of the isolate name indicate identical sequences. Numbers at each node indicate posterior probabilities (PP) greater than 0.80 and bootstrap support (BS) values greater than 50% (PP/BS). Letter X indicates ex-type cultures of each variety.

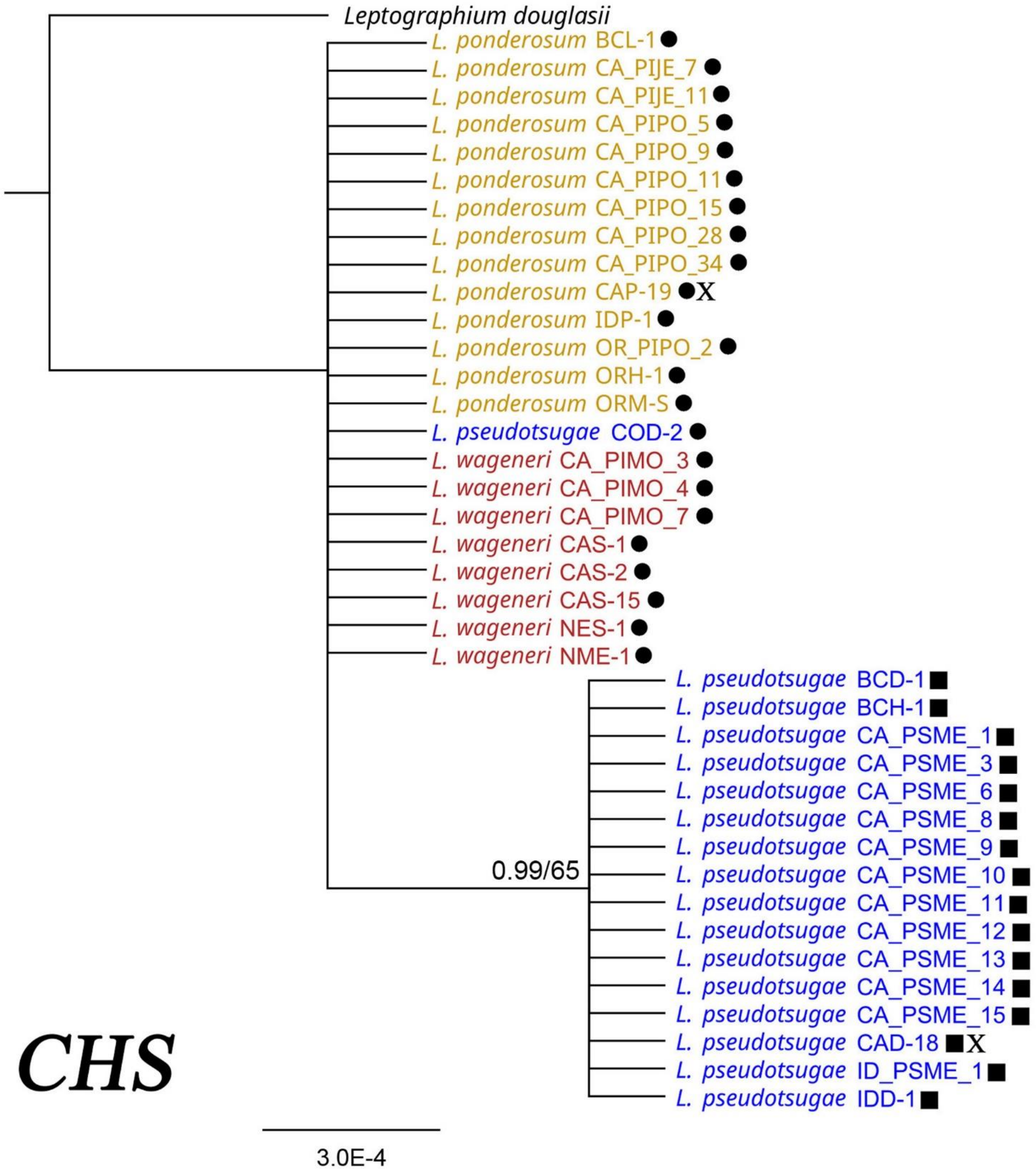

Supplemental Figure 6. Phylogeny of chitin synthase (*CHS*) with *Leptographium douglasii* used as the outgroup. *Leptographium wageneri* (formerly *L. wageneri* var. *wageneri*), *L. ponderosum* (formerly *L. wageneri* var. *ponderosum*), and *L. pseudotsugae* (formerly *L. wageneri* var. *pseudotsugae*) are color-coded in red, yellow, and blue, respectively. The round and square shapes inserted to the right of the isolate name indicate identical sequences. Numbers at each node indicate posterior probabilities (PP) greater than 0.80 and bootstrap support (BS) values greater than 50% (PP/BS). Letter X indicates ex-type cultures of each variety.

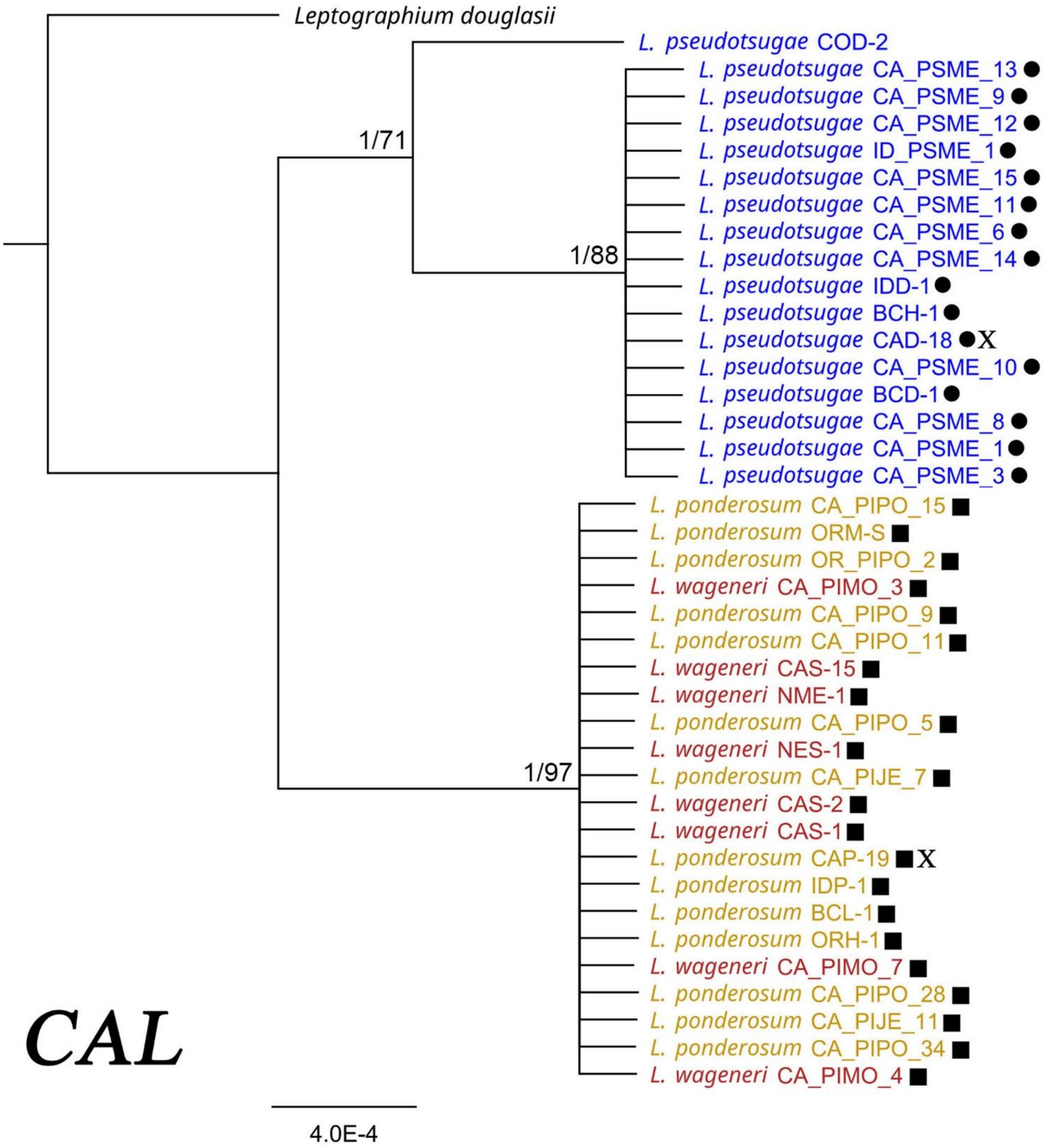

Supplemental Figure 7. Phylogeny of calmodulin (*CAL*) with *Leptographium douglasii* (GenBank accession #KY424522) used as the outgroup. *Leptographium wageneri* (formerly *L. wageneri* var. *wageneri*), *L. ponderosum* (formerly *L. wageneri* var. *ponderosum*), and *L. pseudotsugae* (formerly *L. wageneri* var. *pseudotsugae*) are color-coded in red, yellow, and blue, respectively. The round and square shapes inserted to the right of the isolate name indicate identical sequences. Numbers at each node indicate posterior probabilities (PP) greater than 0.80 and bootstrap support (BS) values greater than 50% (PP/BS). Letter X indicates ex-type cultures of each variety.

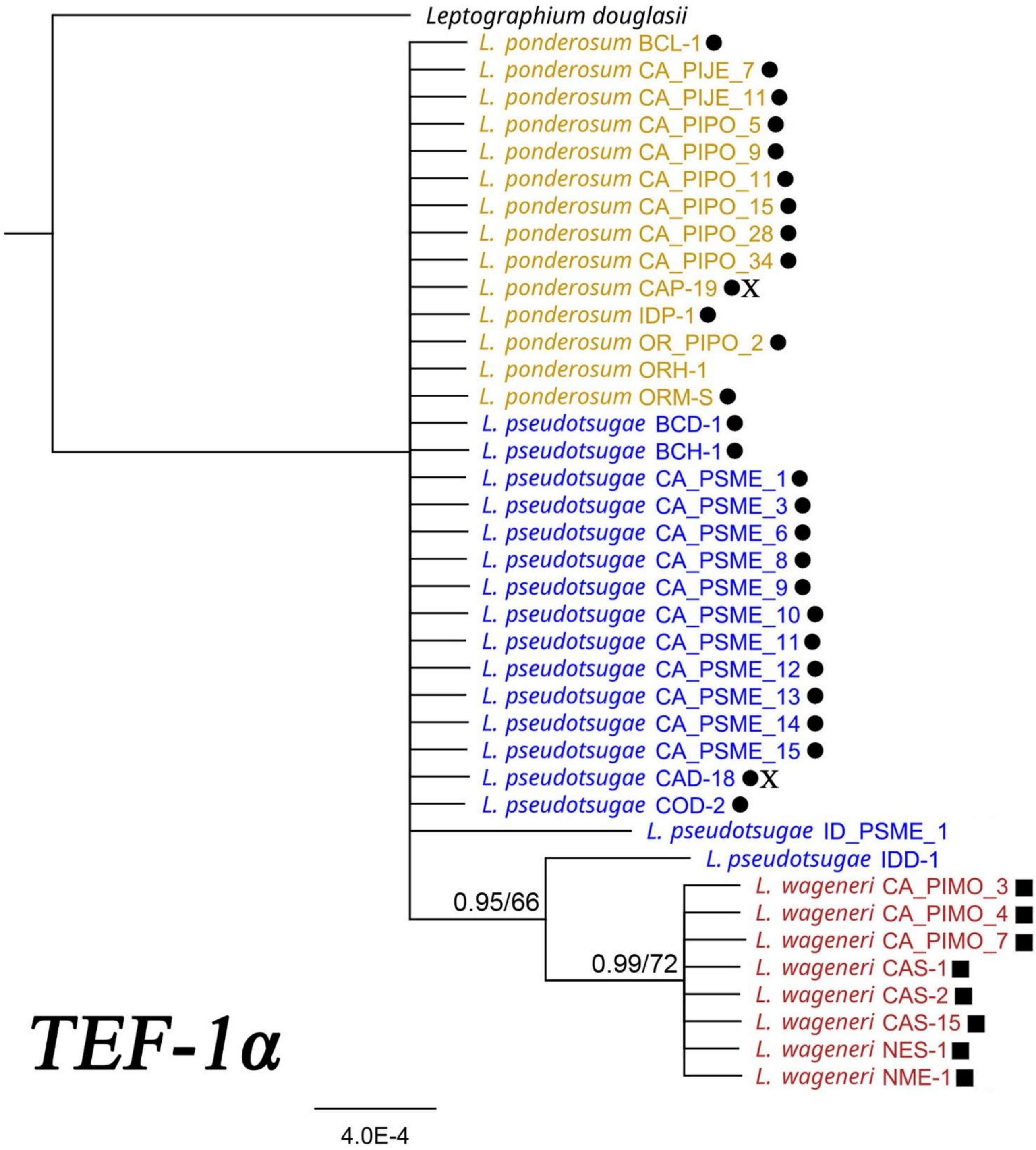

Supplemental Figure 8. Phylogeny of translation elongation factor 1-alpha (*TEF-1α*) with *Leptographium douglasii* (GenBank accession #KY424532) used as the outgroup. *Leptographium wageneri* (formerly *L. wageneri* var. *wageneri*), *L. ponderosum* (formerly *L. wageneri* var. *ponderosum*), and *L. pseudotsugae* (formerly *L. wageneri* var. *pseudotsugae*) are color-coded in red, yellow, and blue, respectively. The round and square shapes inserted to the right of the isolate name indicate identical sequences. Numbers at each node indicate posterior probabilities (PP) greater than 0.80 and bootstrap support (BS) values greater than 50% (PP/BS). Letter X indicates ex-type cultures of each variety.

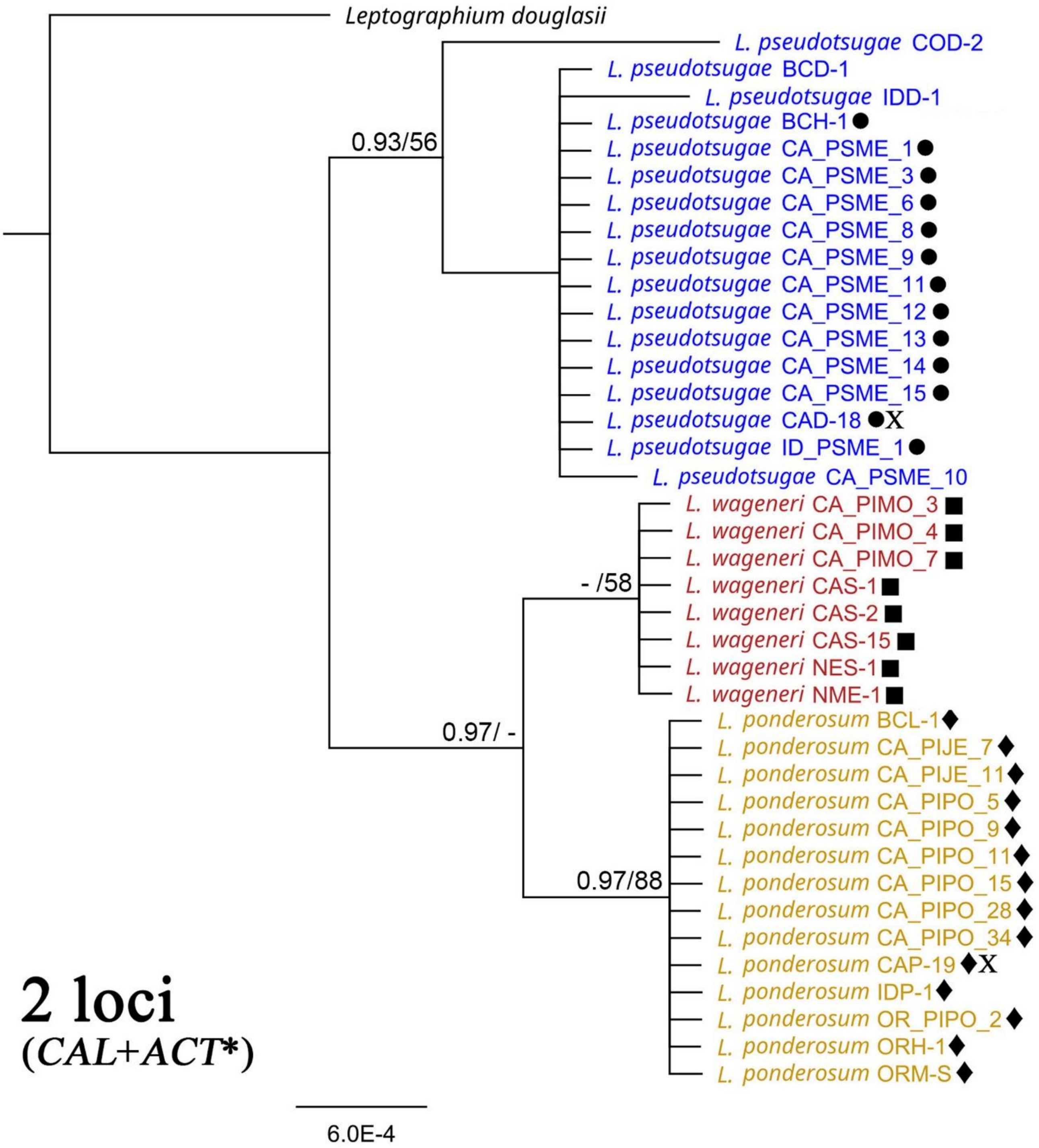

Supplemental Figure 9. Phylogeny trees of the combined calmodulin and actin loci (*CAL*+*ACT**) with *Leptographium douglasii* (GenBank accession numbers, KY424522 and KY424502, respectively) used as the outgroup. *Leptographium wageneri* (formerly *L*. *wageneri* var. *wageneri*), *L. ponderosum* (formerly *L. wageneri* var. *ponderosum*), and *L. pseudotsugae* (formerly *L. wageneri* var. *pseudotsugae*) are color-coded in red, yellow, and blue, respectively. The round, square, and diamond shapes inserted to the right of the isolate name indicate identical sequences. Numbers at each node indicate posterior probabilities (PP) greater than 0.80 and bootstrap support (BS) values greater than 50% (PP/BS). *ACT** indicates actin dataset using Lepact-F & Lepact-R primers. Letter X indicates ex-type cultures of each variety.

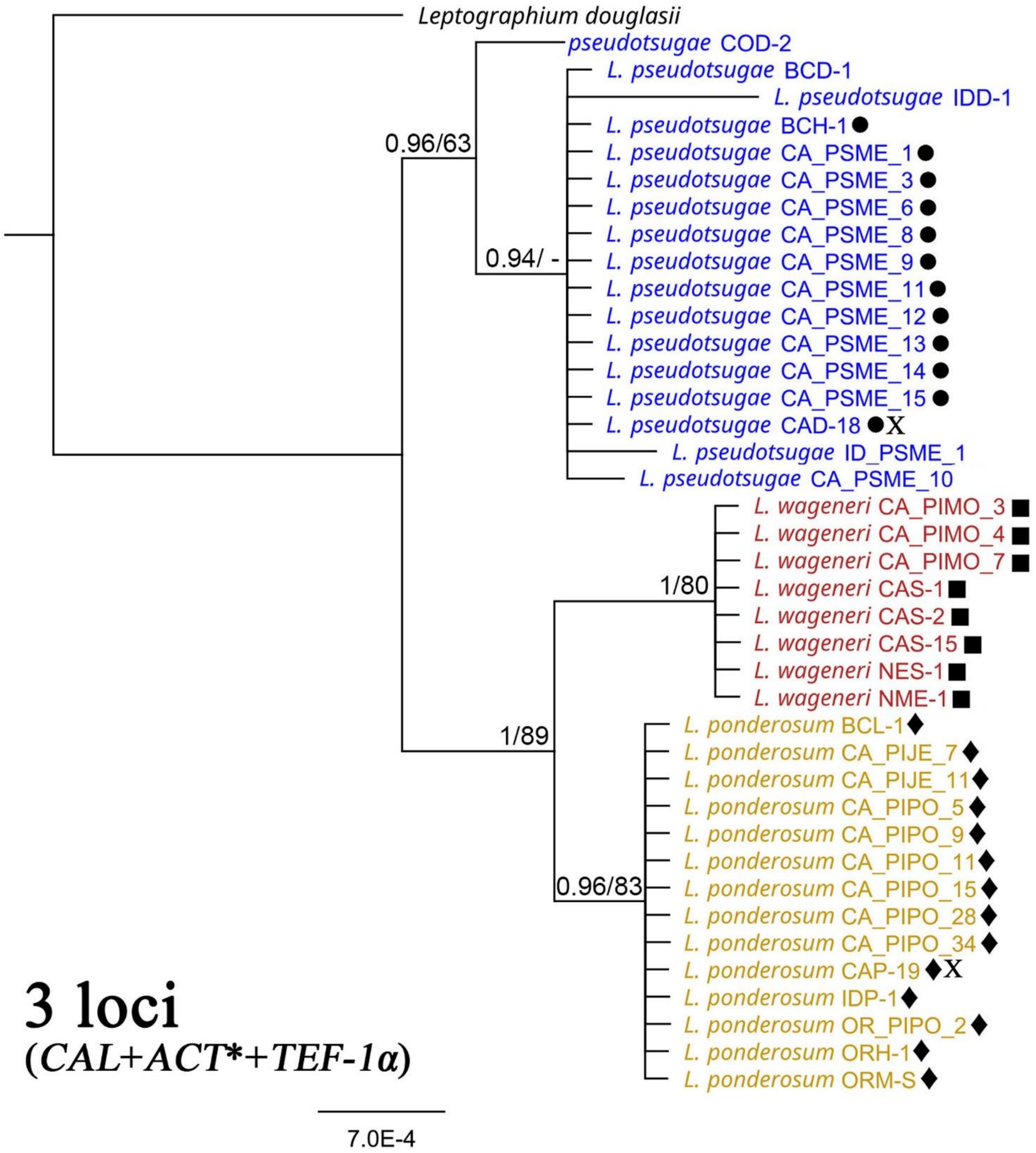

Supplemental Figure 10. Phylogeny of the combined calmodulin, actin, and translation elongation factor 1-alpha (*CAL+ACT**+ *TEF-1α*) loci with *Leptographium douglasii* (GenBank accession numbers, KY424522, KY424502, and KY424532, respectively) used as the outgroup. *Leptographium wageneri* (formerly *L. wageneri* var. *wageneri*), *L. ponderosum* (formerly *L. wageneri* var. *ponderosum*), and *L. pseudotsugae* (formerly *L. wageneri* var. *pseudotsugae*) are color-coded in red, yellow, and blue, respectively. The round, square, and diamond shapes inserted to the right of the isolate name indicate identical sequences. Numbers at each node indicate posterior probabilities (PP) greater than 0.80 and bootstrap support (BS) values greater than 50% (PP/BS). *ACT** indicates actin dataset using Lepact-F & Lepact-R primers. Letter X indicates ex-type cultures of each variety.
